# Modeling Osteocyte Network Formation: Healthy and Cancerous Environments

**DOI:** 10.3389/fbioe.2020.00757

**Published:** 2020-07-22

**Authors:** Jake P. Taylor-King, Pascal R. Buenzli, S. Jon Chapman, Conor C. Lynch, David Basanta

**Affiliations:** ^1^Department of Biology, Institute of Molecular Systems Biology, ETHZ, Zurich, Switzerland; ^2^Mathematical Institute, University of Oxford, Oxford, United Kingdom; ^3^Integrated Mathematical Oncology, H. Lee Moffitt Cancer Center and Research Institute, Tampa, FL, United States; ^4^School of Mathematical Sciences, Queensland University of Technology (QUT), Brisbane, QLD, Australia; ^5^Department of Tumor Biology, H. Lee Moffitt Cancer Center and Research Institute, Tampa, FL, United States

**Keywords:** bone, bone formation, network, mathematical model, osteocyte

## Abstract

Advanced cancers, such as prostate and breast cancers, commonly metastasize to bone. In the bone matrix, dendritic osteocytes form a spatial network allowing communication between osteocytes and the osteoblasts located on the bone surface. This communication network facilitates coordinated bone remodeling. In the presence of a cancerous microenvironment, the topology of this network changes. In those situations, osteocytes often appear to be either overdifferentiated (i.e., there are more dendrites than healthy bone) or underdeveloped (i.e., dendrites do not fully form). In addition to structural changes, histological sections from metastatic breast cancer xenografted mice show that number of osteocytes per unit area is different between healthy bone and cancerous bone. We present a stochastic agent-based model for bone formation incorporating osteoblasts and osteocytes that allows us to probe both network structure and density of osteocytes in bone. Our model both allows for the simulation of our spatial network model and analysis of mean-field equations in the form of integro-partial differential equations. We considered variations of our model to study specific physiological hypotheses related to osteoblast differentiation; for example predicting how changing biological parameters, such as rates of bone secretion, rates of cancer formation, and rates of osteoblast differentiation can allow for qualitatively different network topologies. We then used our model to explore how commonly applied therapies such as bisphosphonates (e.g., zoledronic acid) impact osteocyte network formation.

## Introduction

Advanced cancers commonly metastasize to bone where they often disrupt the normal bone remodeling process (Roudier et al., 2003; Zhang et al., 2013). With the onset of various types of bone cancer, it is common for the bone remodeling process to be disrupted. Much previous work has been focused on macroscopic properties of the resulting bone, e.g., the osteoblastic (net bone formation) and osteolytic (net bone reduction) phenotypes. As bone is formed and resorbed cyclically, osteocyte networks can be morphologically malformed. A relatively unexplored area regarding cancerous bone formation is the study of osteoblast-to-osteocyte differentiation whilst concurrently taking into account network structure. Understanding the full nature of bone formation networks is now becoming extremely important, especially as osteocyte network structure is suspected to limit effectiveness of current anti-cancer therapy (Lerebours and Buenzli, 2016).

Myeloma and (benign) osteoma, osteocytes appear exceptionally spherical with shorter distorted dendrites that are reduced in number (Stinson, 1975; Eisenberger et al., 2008). An experimentally contrasting osteocyte network was observed with unregulated excessive cancerous growth in the presence of osteogenic sarcoma (Stinson, 1975). Broadly speaking, osteocytes within a cancerous microenvironment display either over or under developed phenotypes (see Table 1 and figures in Stinson, 1975) leading to “more connected” or “less connected” networks.

**Table 1:** Cancer type impact on osteocyte topology.

**Cancer type**	**Origin**	**Bone growth**	**Osteocyte topology**	**References**
Osteogenic Sarcoma.	Mesenchymal cells	Mixture	Canaliculi upregulated, Lacunae empty.	Stinson, 1975
Osteoma (benign).	Unknown	Osteoblastic	Canaliculi down regulated, retarded growth.	Stinson, 1975
Myeloma	Plasma cells / White blood cells	Primarily osteolytic	Osteocyte lacunae spherical, canaliculi reduced in number, shorter, distorted.	Eisenberger et al., 2008 Kristinsson et al., 2011
Metastasis (group A)	Lung, Breast, Liver	Primarily osteolytic	Unknown	Eisenberger et al., 2008
Metastasis (group B)	Prostate	Primarily osteoblastic	Unknown	Logothetis and Lin, 2005

Perturbations in osteocyte-network organization can impact both fluid flow and diffusion of metabolites and thereby affect mechanosenzation and signaling (Knothe Tate et al., 2000; Steck and Knothe Tate, 2005; Kerschnitzki et al., 2013; Lerebours and Buenzli, 2016). The exchange of signaling molecules through the lacuno-canalicular network relate to: skeletal unloading, and fatigue damage (Jilka et al., 2013). The range of signaling molecules that have been detected is vast and arise in the regulation of bone mineralization (Hesse et al., 2015), and many other organs^1^. Studies have reported that high-density networks correlate positively with high bone quality (Kerschnitzki et al., 2013).

The functional role of these different network topologies is unclear. Experimental works only reveal a snapshot of the communication between the osteocytes within bone, and the osteoblasts on the bone surface. However, one can use mathematical modeling as a tool for investigation. Marotti et al. suggested that osteoblasts are incorporated into the osteocyte network by mature osteocytes extending their dendrites toward the osteoblast layer (Marotti et al., 1995; Marotti, 2000; Kamioka et al., 2001; Palumbo et al., 2004; Pazzaglia et al., 2010). Experimentally, sclerostin has been stained for and observed within the cancer structures between osteocytes and osteoblasts (Poole et al., 2005). These studies highlight the important roleosteocytes play in osteoblast function and differentiation.

Therapeutically, zoledronic acid is frequently used to treat metastatic breast cancer (BCA) where pathological bone is formed with lower densities of osteocytes per unit volume; zoledronate then helps recover the number of osteocytes by inhibiting osteoclasts—although it is not clear if the network structure is restored.

In this paper, we have developed a stochastic agent-based model to investigate how cancer cells regulate osteocyte behavior and bone formation. We consider osteocyte network formation building on an earlier model of osteocyte generation (Buenzli, 2015), which did not account for network structure. In said model, osteocytes are located within a growing domain that represents the presence of mineralized bone; osteoblasts are located on the surface of the bone substrate. There are two constitutive processes: (*i*) the bone surface moves with a speed proportional to the surface density of osteoblasts; and (*ii*) osteoblasts differentiate into osteocytes. We add to the model by allowing for extra structure relating to the osteocyte's canicular network. We allow for osteocytes to extend dendrites toward the osteoblast layer to signal osteoblast differentiation (we assume these processes occur concurrently). Osteoblasts are also allowed to proliferate and move along the bone surface.

With this model, we aim to link osteocyte density and network structure to biological quantities such as: the rate of osteoid secretion; the rate of osteocyte network formation; and the rate of preosteoblast proliferation. In particular, we investigate how stimulatory or inhibitory network dependent signals influence osteoblast-to-osteocyte differentiation and lead to different osteocyte network properties in newly formed bone.

## 1. Materials and Methods

### 1.1. Histological Analysis of Osteocyte Density

Specimens for analysis were derived from mice that were intratibially inoculated with either saline (Control) or breast cancer (BCa, PyMT cell line) as described previously under University of South Florida IACUC approved protocols (R2238 and R1762-CCL) (Araujo et al., 2014; Tauro et al., 2017). Mice were harvested for analysis prior to breach of the cortical bone (day 21). Separately, BCa-PyMT inoculated mice were treated with a bisphosphonate (zoledronate) over the course of the study period (1mg/Kg, subcutaneously thrice weekly) (Araujo et al., 2014; Tauro et al., 2017). Subsequent to tissue collection and isolation, bones were decalcified, processed and paraffin embedded. sections (5μm) were generated, rehydrated and then stained with either Gomori's Trichrome or Hematoxylin & Eosin using standard procedures.

**Figure 1:**
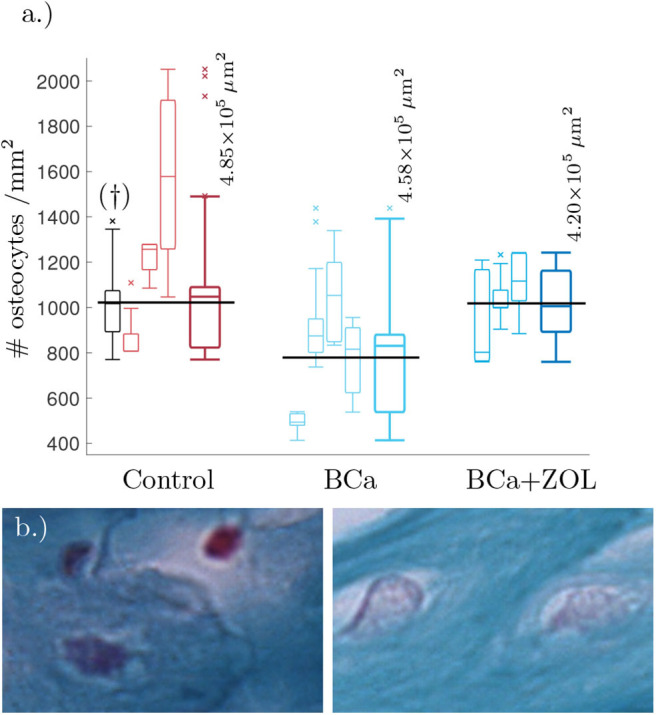
**(a)** Tukey box plot showing results to preliminary image analysis of osteocyte number density. Means plotted in black (see text for details). The far left histogram (marked †) is representative of a collection of histology slides imaged at a lower resolution. **(b)** Comparison between osteocyte sizes: (left) control, (right) bone under breast cancer protocol. Image size corresponds to 32 × 21μm. The smaller box plots are the osteocyte densities for each mouse using 3–8 slides per mouse. Each sample of the osteocyte number density is weighted by the quantity of visible bone area within the sample when determining the data statistics summarized in the plots. The larger box plots show the combined data for mice undergoing the same protocol.

We estimated the area of visible trabecular bone within a pathology image, and subsequently count the number of osteocytes within this region. For full details of this Algorithm, see Appendix A. Unfortunately, the samples were not at a resolution high enough to determine network structure, however it was possible to estimate number of osteocytes per unit area (#osteocytes/mm^2^). These results are presented as box plots in Figure 1.

From Figure 1, breast cancer pathological bone have significant lower osteocyte number densities when compared to healthy bone. Macroscopically, breast cancer is often osteolytic and suppresses osteoblast proliferation and maturation. When applied as therapy, the zoledronate treatment allows for recovery of osteocyte number density.

### 1.2. Mathematical Model

#### 1.2.1. Mathematical Description

**Figure 2:**
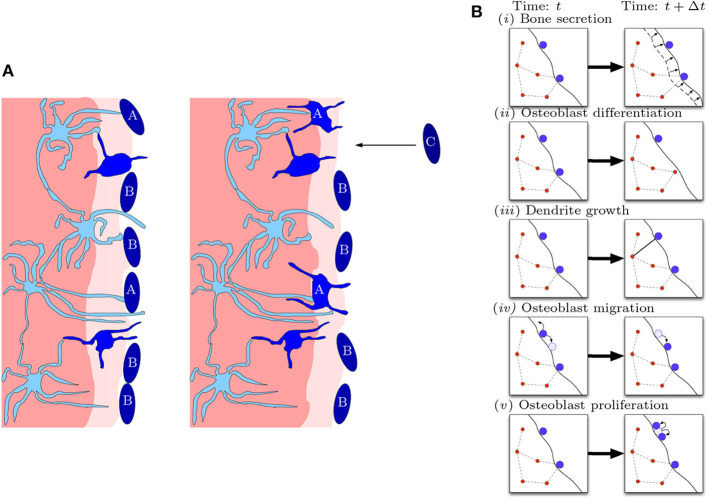
Diagrammatic illustration of bone formation. **(A)** Biological process. Lighter shades of blue indicate more differentiated cells. The lighter shade of pink indicates the deposition front, and the darker shade of pink indicates the mineralization front. The left panel occurs earlier than the right panel. Dendritic osteocytes (light blue) have dendrites that extend toward the osteoblast layer (dark blue). The osteoblasts secrete bone matrix. Osteoblast cells marked with “A” are signaled by the osteocyte network to differentiate into osteocytes. Osteoblast cells marked with “B” do not differentiate and stay on the outer bone surface. Osteoblast cells marked with “C” arrive at the bone front after differentiating from precursor osteoblasts (preosteoblasts) (this figure is adapted from Franz-Odendaal et al., 2006). **(B)** Model representation of biological process. In a small time step, the following events can occur: (i) bone secretion; (ii) osteoblast differentiation; (iii) dendrite growth; (iv) osteoblast migration; and (v) osteoblast proliferation.

Our model is a stochastic agent-based model for the bone formation phase only. The agents in our model are osteocytes and osteoblasts occupying nodes within a spatial network. The osteoblast-to-osteocyte differentiation pathway is subdivided into eight phenotypic stages: (*i*) preosteoblast; (*ii*) preosteoblastic osteoblast; (*iii*) osteoblast; (*iv*) osteoblastic osteocyte; (*v*) osteoid-osteocyte (Type II preosteocyte); (*vi*) Type III preosteocyte; (*vii*) young osteocyte; and (*viii*) old osteocyte (Franz-Odendaal et al., 2006). Additionally, the secretion of bone occurs as two steps: first osteoid is deposited as a collageneous scaffold, and then mineralization occurs to confer strength. Stages (*iv*)–(*vi*) are cells after the deposition front but before the mineralization front, surrounded by a non-mineralized osteoid matrix (i.e., there is scaffold around them). Stages (*vii*)–(*viii*) are cells whose volume has depleted (endoplasmic reticulum and Golgi apparatus reduction) and are in mineralized bone. The diagram in Figure 2 shows the bone-formation step. Here we are only interested in the structure of a mature osteocyte network [stages (*vi*)–(*viii*)], so we avoid modeling the full biology intricacy (e.g., cell sub-classifications, proteins etc) for simplicity. We model mobile preosteoblasts (disconnected from the osteocyte network) in front of the bone deposition front that proliferate; stationary mature osteoblasts that secrete osteoid and are connected to the osteocyte network; and osteocytes that form dendrites with mature osteoblasts.

In our model, the spatial network has connections, between cells, representing the ability for two cells to communicate; physically this communication is mediated through dendrites.

The model consists of the following processes: (*i*) bone secretion; (*ii*) osteoblast differentiation; (*iii*) dendrite growth; (*iv*) preosteoblast migration; and (*v*) preosteoblast proliferation (see Figure 2). Agents of the same type (osteocytes or osteoblasts) follow the same rules, although each agent may have different properties, e.g., position, number of connections, in addition to type.

Bone secretion is carried out by osteoblasts secreting bone in a small region around themselves, orthogonal to the bone surface (in the normal direction). Osteoblasts can also become buried and differentiate into osteocytes. The rate of osteoblast differentiation may depend on if the osteoblast in question is in communication with the osteocyte network in bone.

For dendrite growth, osteocytes and osteoblasts create a communication channel (i.e., become connected in the network) at a rate that is a function of the distance between the two cells. Our model is consistent with the suggestions in Palumbo et al. (1990) that dendrites grow away from osteocytes within bone and toward the osteoblast layer on the bone surface.

Movement and cell division are included for pre-osteoblasts that are disconnected from the osteocyte network. Pre-osteoblasts move along the bone surface by means of a diffusive process; they are also able to proliferate and divide into create two daughter cells.

Our simulations are carried out in two dimensions, but they represent a slice from a three dimensional organ, in which the third dimension *L*_*z*_ is the typical distance between osteocyte centers (*L*_*z*_ ≈ 40μm), projected onto two dimensions (see Figure 7 in Appendix). The *x*-direction is the main direction of bone growth, and the volume occupied by the bone increases in time. We impose periodicity in the *y*-direction (orthogonal to bone growth) to avoid boundary effects.

In all the simulations in this paper, we consider bone formation after a cement line has been deposited. A cement line is a 1-5μm region of hypermineralized (and collagen deficient) bone (Skedros et al., 2005) is deposited after bone resorption to prepare surfaces for new bone formation. When this cement line is deposited, osteocytes from deep within the bone are not necessarily in communication with osteoblasts on the other side of the cement line (Qin et al., 1999).

**Figure 3:**
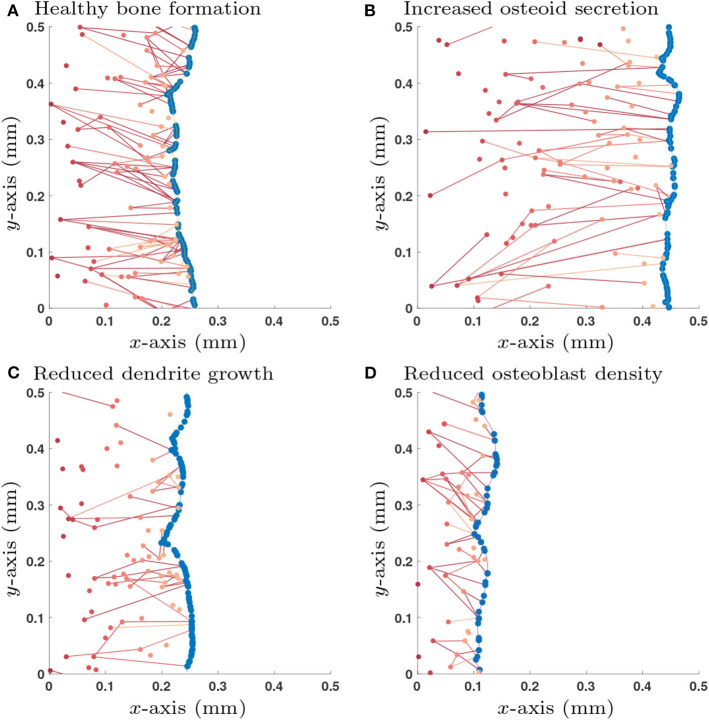
Single simulation runs of bone growth with different parameter choices: **(A)** Healthy parameter set (as shown in **Table 3**); **(B)** Increased osteoid secretion (η → 2η); **(C)** Reduced dendrite growth (α → α/2); **(D)** Reduced osteoblast surface density ($\tilde{p}\to \tilde{p}/2$). Osteoblast are colored in blue and osteocytes (and their network connections) are in red; darker shades of red denote osteocytes that were buried earlier in time. All simulations are shown after 365 days.

Accordingly, we use the initial condition that there are no old osteocytes to communicate with (no initial network structure at the onset of bone formation) and there is an initial surface density of 6 × 10^3^mm^−2^ pre-osteoblasts that do not have any network structure. We also specify that once an osteoblast is buried, a new osteoblast takes its place (so the total number of osteoblasts at any time is constant). This new osteoblast has no network structure. Therefore, one can interpret this configuration of the model as the scenario in which pre-osteoblasts are in abundance at the bone interface. New osteoblasts then move into the cell gaps on the bone surface as space becomes available (Figure 3).

#### 1.2.2. Simulation and Analysis

Individual simulation runs of our stochastic agent-based model were performed with a fixed time step Monte Carlo algorithm (see Appendix B). However, a single realization is unrepresentative of the stochastic process, so many simulations must be carried out to work out statistics of the process along with parameterization. This can be computationally demanding, especially as the system grows in size.

As an alternative to large numbers of simulations, we can utilize mean field equations. We have shown previously (Taylor-King et al., 2017) that in the limit as the number of nodes in the network increases, one is able to derive a mean-field partial integro-differential equation for the expected number of nodes at a particular position in the domain connected to a fixed number of cells. By solving the mean-field equations, one can also calculate the degree distribution of the nodes of the network. The validity of the mean-field assumption is discussed in Taylor-King et al. (2017).

Simulations of the stochastic mathematical model suggest that the system is approximately homogeneous in *y*, the direction orthogonal to bone growth (see Figure 3). Under the assumption of homogeneity in *y*, the mean-field equations depend only on *x*, the direction of bone growth. The solutions of these equations provide spatial profiles of the densities of osteocytes and their network structure perpendicularly to the bone surface (see Appendix C).

Our mean-field equations take the form of two coupled hierarchies of integro-partial differential equations (see Equations 23–24 in Appendix). These equations can be solved to indicate how many particles one could expect to find within a small region of space are, connected to *k* other cells (referred to as degree *k*) at time *t*. We denote by *v_k_* the surface density of osteoblasts (number per unit area, [*v_k_*] = mm^−2^) connected to *k* osteocytes on the bone surface; and *w_k_* the number density osteocytes (number per unit volume, [*w_k_*] = mm^−3^) connected to *k* osteocytes or osteoblasts with position *x* at time *t* within the bone. Therefore, we write the total density of all osteoblasts (regardless of degree) as $p=\underset{k}{\sum }vk$ and total density of osteocytes (regardless of degree) as $q=\underset{k}{\sum }w_{k}$. The average number of osteocytes connected to an osteoblast is the average degree of osteoblasts, i.e.,

(1) $$\begin{array}{r} \langle k\rangle \text{Ob}=\frac{\overset{\infty }{\underset{k=0}{\sum }}kvk}{p}; \end{array}$$

and the average number of cells (osteoblasts and osteocytes) in communication with an osteocyte is the average degree of osteocytes, i.e.,

(2) $$\begin{array}{r} \langle k\rangle \text{Ot}=\frac{\overset{\infty }{\underset{k=0}{\sum }}kwk}{q}. \end{array}$$

The full equations for *v_k_* and *w_k_* are derived in Appendix C and solved in Appendix D. In the present work, we only consider an uninterrupted bone formation process so that the density of osteocytes *q* at point *x* corresponds to that generated by the terminal differentiation of osteoblasts when the bone deposition front was at *x*.

The mean-field equations admit a traveling wave solution, corresponding to steady bone growth if the osteoblast proliferation rate is such that the surface density of osteoblasts is constant. Such a solution is useful both to explain the model and to investigate the qualitative effects of perturbations to parameters. In the following, we denote the traveling wave solution with a tilde.

## 2. Results

### 2.1. Selection of Differentiation Mechanism

We wish to investigate the effects of different model choices for the rate of osteoblast-to-osteocyte differentiation, *D_k_*.

For a given surface density of osteoblasts, the volumetric density of inclusions embedded in a tissue during bone formation is determined by two dynamic processes: the rate of osteoblast terminal differentiation and the tissue growth rate (Buenzli, 2015). If the rate of osteoblast terminal differentiation is identical for all osteoblasts at a given location, i.e., *D_k_* is independent of *k* ($Dk\equiv \hat{D}$), the density of osteocytes generated is given by Buenzli (2015)

(3) $$\begin{array}{r} \tilde{q}\equiv \frac{\hat{D}\tilde{p}}{\tilde{\nu }}=\frac{\hat{D}}{\kappa \text{form}}, \end{array}$$

where $\hat{D}$ is the terminal differentiation rate, i.e., the probability per unit time for a single osteoblast to become embedded as an osteocyte, $\tilde{\nu }=\kappa \text{form}\tilde{p}$ is the normal velocity of the bone interface, and κform is the rate of bone deposition per osteoblast. If the rate of osteoblast terminal differentiation depends additionally on the number of connections *k* they have with osteocytes, the density of osteocytes generated sums up the contributions of all *k*-degree osteoblasts

(4) $$\begin{array}{r} \tilde{q}\equiv \overset{\infty }{\underset{k=0}{\sum }}\frac{Dk\tilde{v}k}{\tilde{\nu }}. \end{array}$$

In contrast to Equation (3), the density of osteocytes given by Equation (4) depends explicitly on the population of osteoblasts, through the proportions $vk/\tilde{p}$ of *k*-degree osteoblasts. Notice that this expression determines the density of osteocytes created at the moving bone deposition front. It does not account for processes that may subsequently affect osteocyte density such as osteocyte apoptosis, bone resorption and remodeling, which may remove and replace osteocytes subsequently (Lerebours and Buenzli, 2016).

To explore different differentiation mechanisms we alter both: the rate of dendritic growth, α, and the mechanism behind the rate of osteoblast differentiation, *D_k_*.

#### 2.1.1. Assuming No Network Influence: Degree-Independent-Rate Model

To account for the potential of network independent differentiation we assume that the number of osteocytes each osteoblast is in communication with does not impact the rate of osteoblast differentiation; we write

(5) $$\begin{array}{r} D\left(\text{null}\right)k\equiv \hat{D}, \end{array}$$

for all values of *k* = 0, 1, …, ∞.

After parameterizing the system using experimental measurements found from literature (see **Table 3**), with this choice of osteoblast differentiation there remains two undefined (free) parameters in the model: the rate of dendrite growth $\hat{\alpha }$, and the rate of osteoblast differentiation $\hat{D}$; and we calibrate these parameters based on two further experimental observations: the mean degree of an osteoblast connectivity $\langle \tilde{k}\rangle \text{Ob}=1$ (see Appendix E.6); and the number density of osteocytes $\tilde{q}=2.375\times 10^{4}{\text{mm}}^{-3}$. By carrying out mathematical analysis in the traveling wave regime, we obtain formulas linking these quantities together (see Appendix D.2). We determine parameters as $\hat{\alpha }=1.39\times 10^{-3}{\text{day}}^{-1}$ and $\hat{D}=2.59\times 10^{-3}{\text{day}}^{-1}$.

If one changes the rate of osteoblast-to-osteocyte differentiation, $\hat{D}$, we observe a linear relationship with the number density of osteocytes buried, $\tilde{q}$ (see Equation 3). Changing the rate of formation, $\hat{\alpha }$, leads to a linear relationship with the mean osteoblast degree, $\langle \tilde{k}\rangle \text{Ob}$, and the mean osteocyte degree, $\langle \tilde{k}\rangle \text{Ot}$. These effects are decoupled — changing the rate of osteoblast-to-osteocyte differentiation, $\hat{D}$, has no effect on the network structure, and changing the rate of dendrite formation, $\hat{\alpha }$, has no effect on the osteocyte density, $\tilde{q}$. This will not be the case when osteoblast terminal differentiation is coordinated by the osteocyte network.

The assumption that osteoblast differentiation is independent of the osteocyte network is unlikely. First, as reviewed in Gohel et al. (1995), osteoblasts can be signaled by osteocytes to adhere to the mineral matrix and grow dendrites (subsequently differentiating) via the insulin-like growth factor 1 (IGF-1). Second, sclerostin secreted by osteocytes has been shown to act briefly as an inhibitory signal to prevent excessive osteoblast differentiation and allowing for coordinated osteocyte network formation (Poole et al., 2005). Note that in Poole et al. (2005), sclerostin was stained for and observable within the osteocyte's dendritic protrusions. These experiments, along with the three-dimensional scans taken by Kamioka et al. (2001), strongly suggest that osteocytes signal osteoblast differentiation through the extension of dendrites toward the osteoblast layer.

#### 2.1.2. Modeling Network Effects

We now consider a range of models in which the pre-existing osteocyte network can either have an stimulatory or inhibitory effect on osteoblast differentiation. We consider differentiation rates of the form

(6) $$\begin{array}{r} Dk=\left\{ \begin{array}{ccc} \lambda & \text{if} & k=0,\\ \lambda +f\left(k\right) & \text{if} & k\geq 1, \end{array}\right. \end{array}$$

where λ is the network-independent rate of osteoblast differentiation and *f* = *f*(*k*) is the contribution to osteoblast differentiation for an osteoblast connected to *k* osteocytes. When *f* > 0 the network has an excitatory effect on osteoblast differentiation, and when *f* < 0 the network has an inhibitory effect on osteoblast differentiation. To prevent negative differentiation rates, we require that

(7) $$\begin{array}{r} \lambda >0,\text{and}\lambda +f\left(k\right)\geq 0,\;\forall k\geq 1. \end{array}$$

In the main text (see section 2.1.3 below), we consider only the case where *f* is a constant, *f* = γ. Other choices of *f* are considered in Appendix E, i.e., i.e., cumulative activation (*f*∝*k*) and diminishing activation (*f*∝1/*k*). It should be noted that choices of *f* that have non-monotonic behavior (i.e., a local maximum/minimum exists) can lead to non-monotonic profiles in *q*.

#### 2.1.3. Proposed Mechanism: Switch-Like Influence

Switch-like mechanisms are frequently found in biology (Cherry and Adler, 2000); at a cellular level this includes initiating mechanisms for proliferation and differentiation (Xia et al., 2006). For a switch-like osteoblast differentiation, we take

(8) $$\begin{array}{r} D\left(\text{swt}\right)k=\left\{ \begin{array}{ccc} \lambda \text{swt} & \text{if} & k=0,\\ \lambda \text{swt}+\gamma \text{swt} & \text{if} & k\geq 1. \end{array}\right. \end{array}$$

If the osteoblast on the bone surface is not in communication with any other osteocytes (*k* = 0) then it has an network-independent differentiation rate λ_swt_. If osteoblasts are in contact with one or more osteocytes, then there is an induced differentiation rate λ_swt_+γ_swt_ where γ_swt_ is the added contribution from the dendritic network.

Our technique for parameter identification with this family of terminal differentiation rates is based on the mean-field equations. We choose a network independent component of osteoblast differentiation such that $\lambda \text{swt}\neq \hat{D}$, leaving 2 free parameters αswt and γ_swt_. We then determine the free parameters by imposing that $\langle \tilde{k}\rangle \text{Ob}=1$ and $\tilde{q}=2.375\times 10^{4}{\text{mm}}^{-3}$ in the traveling wave regime. By making these assertions, if $\lambda \text{swt}<\hat{D}$, the network contribution will always have an stimulatory effect on osteoblast differentiation; and if $\lambda \text{swt}>\hat{D}$, then the network contribution will always inhibit osteoblast differentiation.

To investigate the role of intrinsic to extrinsic osteoblast differentiation rates, we assume that the network-independent differentiation rate λ_swt_ contributes half the total rate of osteoblast-to-osteocyte differentiation rate of the null model (so $\lambda \text{swt}=\hat{D}/2$). We then determine that αswt = 2.08 × 10^−3^day^−1^ and γ_swt_ = 2.59 × 10^−3^day^−1^. Another option would be for an inhibitory contribution, in which case we can set $\lambda \text{swt}=3\hat{D}/2$, and then αswt = 6.96 × 10^−3^day^−1^ and γ_swt_ = −2.60 × 10^−3^day^−1^.

#### 2.1.4. Differentiation Mechanism Comparison

**Figure 4:**
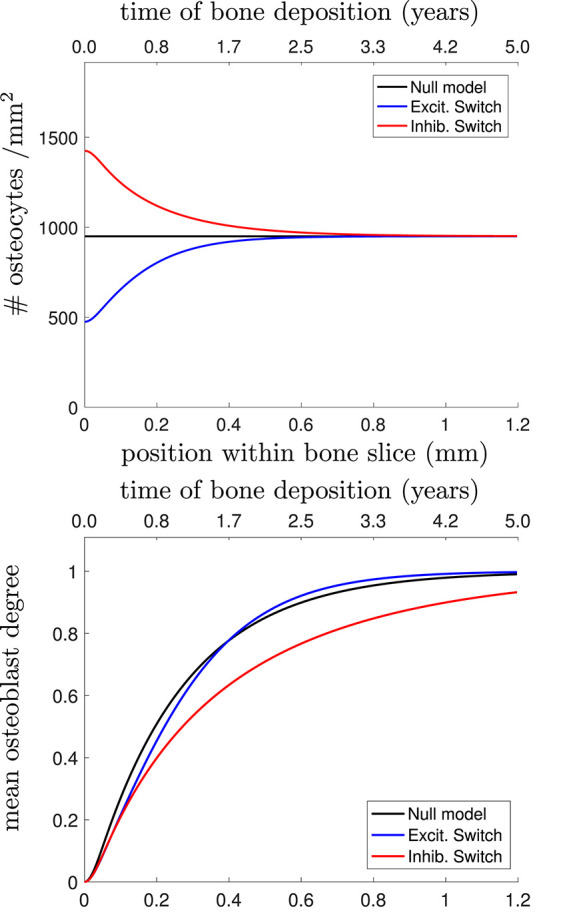
**(Top)** Osteocyte density profile [$q\left(t,x=\tilde{\nu }t\right)$], **(Middle)** mean osteoblast degree over time [${\langle k\left(x=\tilde{\nu }t\right)\rangle }_{\text{Ob}}$], and **(Bottom)** mean osteocyte degree over time [${\langle k\left(x=\tilde{\nu }t\right)\rangle }_{\text{Ot}}$] when solving Equations (23)–(25) in Appendix. The black line shows the null model, the blue line shows the stimulatory switch model, and the red line shows the inhibitory switch model.

In Figure 4, we plot the osteocyte density profile, the mean osteoblast degree over time, and the mean osteocyte degree over time using Equations (23)–(25) in Appendix for 3 proposed choices of *D_k_*: the null model, stimulatory network contributions, and inhibitory network contributions.

Figure 4 shows that in all 3 models, it takes approximately 1 year (~0.7mm) to get to the steady-state desired osteocyte density. We see similar results for the mean osteoblast degree, except that inhibitory contributions to osteoblast differentiation require more time for the osteoblasts to reach the steady-state degree distribution. The main means for differentiation between the three models is the resulting mean osteocyte degree profile. The osteocyte density profile also differs at the onset, but slowly relaxes to the steady state profile. Keeping $\tilde{p}$, $\tilde{q}$, $\langle \tilde{k}\rangle \text{Ob}$ fixed, stimulatory network contributions to osteoblast differentiation leads to more connected osteocyte networks with initially low numbers of osteocytes (higher final value of 〈*k*〉Ot), and inhibitory network contributions lead to less connected networks with initially high numbers of osteocytes (lower final value of 〈*k*〉Ot).

Capturing network structure and choosing a model that best reflects reality is difficult. For instance, the mean osteoblast degree profile takes a long period of time before settling to the steady-state mean osteoblast degree of $\langle \tilde{k}\rangle \text{Ob}=1$. One assumption we have made throughout our model selection process was that the osteoblast surface density was approximately constant. It may be the case that this surface density changes over time (Eriksen et al., 1984; Parfitt et al., 1987). Were it the case that the surface density of osteoblasts was initially higher density before decreasing it would speed up the timescales until a steady-state traveling wave profile was reached.

### 2.2. Parameter Analysis

We explored a wide range of model parameters to further understand their impact on osteocyte network topology. For the switch-like model in section 2.1.3, we use the traveling wave analytic expressions described in Appendix D.2 to investigate how dependent variables $\tilde{q}$, $\tilde{\nu }$, $\langle \tilde{k}\rangle \text{Ob}$, and $\langle \tilde{k}\rangle \text{Ot}$ change due to a single perturbation in one of the independent variables η, $\tilde{p}$, κdiff, λ, γ and α, i.e., a sensitivity analysis, see Table 2. In addition to the dependent variables, we also include the number of dendrites per unit area $\tilde{M}=\tilde{q}\langle \tilde{k}\rangle \text{Ot}/2$ which is a quantity that could be determined experimentally. Depending on whether osteocytes activate or inhibit the rate of osteoblast differentiation, we obtain different results for parameters λ, γ and α. For visualization purposes, we show a few stochastic simulation runs for a few specific examples in Figure 3; these examples use the switch-like model in described in section 2.1.3 with the inhibitory parameter configuration ($\lambda \text{swt}=3\hat{D}/2$, αswt = 6.96 × 10^−3^day^−1^, γ_swt_ = −2.60 × 10^−3^day^−1^).

**Table 2:** Prediction summary.

**Increased parameter (↑)**	**Symbol**	**$\tilde{q}$**	**$\tilde{\nu }$**	**$\langle \tilde{k}\rangle \text{Ob}$**	**$\langle \tilde{k}\rangle \text{Ot}$**	**$\overset{\sim }{M}$**
Rate of bone secretion	η	↓	↑	↓	↓	↓
Surface osteoblast density	$\tilde{p}$	−	↑	−	−	−
Osteoblast migration speed	κdiff	−	−	−	−	−
**Excitatory switch model configuration**.						
Network-independent rate of osteoblast differentiation	λ	↑	−	↑	−	↑
Network-dependent rate of osteoblast differentiation	γ	↑	−	↓	−	↑
Rate of dendrite growth	α	↑	−	↑	↑	↑
**Inhibitory switch model configuration**.						
Network-independent rate of osteoblast differentiation	λ	↑	−	↓	−	↑
Network-dependent rate of osteoblast differentiation	γ	↑	−	↓	−	↑
Rate of dendrite growth	α	↓	−	↑	↑	↑

*In the leftmost column, we list parameters that are to be increased. We use the notation that (↑) denotes an increase, (↓) denotes a decrease and (−) signifies no change. Decreasing the parameters in the leftmost column will reverse the directions of the arrows*.

Osteocyte density is mostly determined by the secretory rate and terminal differentiation rate, with some weak osteoblast density dependence via the frequency distribution of osteoblast degrees via equation (4). Table 2 confirms this as $\tilde{q}$ is dependent on the secretory rate (η) or the terminal differentiation rate (λ, γ, α), but not osteoblast density $\tilde{p}$. Contrasting these results to those given in Figure 4 and Table 2 shows intra-model variability by perturbing parameters, but Figure 4 shows inter-model differences by changing the mechanism for osteoblast differentiation.

Whilst many parameters change the density of osteocytes, only the rate of dendrite growth α and the rate of bone formation η can change the mean osteocyte degree in the steady state regime. A counter intuitive result also specifies that altering the rate(s) of osteoblast differentiation (parameters λ_swt_ and γ_swt_) changes the osteocyte density (variable $\tilde{q}$) and the osteoblast degree (variable $\langle \tilde{k}\rangle \text{Ob}$), but not the degree of these newly formed osteocytes (variable $\langle \tilde{k}\rangle \text{Ot}$). This is possible because osteoblasts are constantly being replaced in the steady state regime; therefore parameters λ_swt_ and γ_swt_ are changing the osteocyte density by modifying the mean time osteoblasts secrete bone before differentiation — without changing the mean osteocyte degree.

As we mentioned in 2.1.1, inhibitory network contributions to osteoblast differentiation is more likely in the presence of sclerostin, but stimulatory network contributions may also be possible via IGF-1. Determining which of these two mechanisms drives osteoblast differentiation will require further experimental work.

## 3. Discussion

We have presented a model for the formation of an osteocyte network and identified parameters for healthy bone formation. By perturbing parameters, one can investigate irregular bone formation and the resulting osteocyte topology changes. One can also then predict the driving differentiation markers that osteoblasts exhibit that would lead to these morphological changes. In the context of zoledronate therapy for breast cancer, we have used our model to propose how this commonly used clinical treatment impacts bone formation. For future experiments, we have suggested how measurable quantities link to underlying mechanisms.

The model proposed has some limitations, one could suggest many improvements to the model to improve our idealization of the osteocyte network; we discuss some of these below. Additionally, one might also want to consider the inclusion of chemical species representing proteins of interest, and the inclusion of osteoclasts to incorporate bone resorption. However, our model acts as a first step toward mathematically modeling osteocyte network formation, and avoids making overly specific assumptions on underlying mechanisms.

We now comment on how various aspects of our model compare to biological reality.

### 3.1. General Implications for Cancerous Bone Growth

A number of previous mathematical models have examined osteocyte density, but none of them have explored network structure. Graham et al. (2013), Moroz et al. (2006), and Wimpenny and Moroz (2007) give ordinary differential equation (non-spatial) models for cell populations; these include osteoblast, osteoclast, and osteocyte populations. Existing models of healthy bone remodeling (homeostasis) include spatiotemporal models (Ryser et al., 2009, 2010; Buenzli et al., 2012), but do not explicitly include osteocyte generation; these models have been adapted for the cancerous regime in Ryser et al. (2012). For TGFβ targeted therapy, modeling approaches have been used to optimize the treatment window of application (Cook et al., 2016). Mechanical focused methods capturing stresses and strains on the bone have also been explored (Rejniak and Anderson, 2011; van Oers et al., 2014). A general continuum modeling approach was also proposed in Buenzli (2015).

Our model-derived results show that osteocytes are either over differentiated (excessive dendrite growth) or underdeveloped (diminished dendrite growth). Additionally, we show that the osteocyte number density tends to decrease.

With experimental data that gives information on network structure (e.g., transmission electron microscopy, India ink histology stains), one should be able to approximately measure at least 2 of 3 quantities: the number of osteocytes present (quantitative estimate); whether the osteocytes are over-differentiated or underdeveloped (qualitative estimate); and finally the density of dendrites (quantitative estimate). Therefore, we should be able to compare a pathological bone slide to a (healthy) control slide and determine differences between: the osteocyte number density (*q*); the mean osteocyte degree ($\langle \tilde{k}\rangle \text{Ot}$); and the density of dendrites ($\tilde{M}$).

Furthermore, our model also makes testable predictions in regards to:

- If all 3 quantities (osteocyte number density, mean osteocyte degree, and dendrite area) have increased (resp. decreased), this corresponds to either: osteoblasts on the bone surface producing too little (resp. too much) osteoid when compared healthy bone; or that the rate of dendrite growth has increased (resp. decreased) in the excitatory switch model configuration.
- If the osteocyte number density has increased (resp. decreased) with an opposing decrease (resp. increase) in $\langle \tilde{k}\rangle \text{Ot}$ or $\tilde{M}$, then this must correspond to the rate of dendrite growth changing but in the inhibitory switch model configuration.
- If the osteocyte density increases (or decreases), but the mean osteocyte connectivity remains constant, our model suggests this relates to a change the rate of osteoblast differentiation.

### 3.2. Implications for Zoledronate Treatment

Breast cancer is known to be osteolytic by promoting osteoclast mediated bone destruction. When applied as a therapy for breast cancer, the zoledronate treatment slows down bone resorption (along with other effects, Polascik and Mouraviev, 2008). This treatment is also associated with a recovery of osteocyte number density, see Figure 1.

Changing the proliferation ability of osteoblasts in the model changes the osteoblast surface density. This changes the quantity of bone produced per unit time, but not the network structure or steady state osteocyte density. In our model, changing the process of osteoblast/osteocyte maturation corresponds to changing either: the mechanism behind osteoblast differentiation (parameters λ, γ), or changing the rate of dendrite growth (parameter α).

In breast cancer (BCa), osteocytes have fewer dendritic connections to other osteocytes and osteocyte density is lower. Therefore the mean osteocyte degree of connectivity is reduced. To achieve a simultaneous decrease in osteocyte density and mean osteocyte degree in the model, one would either have to: decrease the rate of dendrite growth in an stimulatory switch model configuration; or increase the rate of bone secretion per osteoblast.

Given that the zoledronate treatment restores osteocyte density, we propose that future studies investigate how zoledronate acts on the osteoblast-to-osteocyte differentiation pathway. If bone treated with zoledronate has a healthy osteocyte network present, then one can assume zoledronate also targets (and restores) the rate of dendrite growth. However, if bone treated with zoledronate has a different network topology, one can conclude the differentiation mechanism is targeted, i.e., the network-independent or network-dependent rate of osteoblast differentiation has changed.

#### 3.2.1. Triggering Osteoblast Differentiation

During a bone remodeling event, the total number of osteoblasts generated is far larger than the total number of new osteocytes generated (Parfitt, 1994). Pazzaglia et al. (2014) have estimated that only 1 in 67 osteoblasts become embedded in bone matrix as osteocytes over the depth of a single osteocyte. In our model using the steady state regime with the parameters from Table 3, over a length-scale of 5μm approximately 1 in 50 osteoblasts achieve terminal differentiation. The exact mechanisms behind this process are still poorly understood; they may involve physical processes such as burial by neighboring osteoblasts, or self-burial (Franz-Odendaal et al., 2006). It has also been suggested that subpopulations of osteoblasts are predestinated to become osteocytes (Marotti et al., 1992), and that this selection may be determined by the number of connections with osteocytes (Kamioka et al., 2001).

**Table 3:** Model parameters, see Appendix E for more details.

**Parameter name**	**Symbol and value**	**Units**	**References**
**General parameters**			
Osteoblast diffusion constant	κdiff = 4.5 × 10^−5^	mm^2^day^−1^	Araujo et al., 2014
Domain size	*L*_*y*_ = 0.5, *L*_*z*_ = 0.04.	mm	−
Bone secretion rate parameter	η = 7.27 × 10^−5^	mmday^−1^	Araujo et al., 2014
Dendrite growth shape parameter	β = 25 × 10^−3^	mm	Hannah et al., 2010
Bone secretion shape parameter	ι = 15 × 10^−3^	mm	Araujo et al., 2014
**Traveling wave parameters**			
Traveling wave speed (rate of bone formation)	$\tilde{\nu }=0.656$	μmday^−1^	Araujo et al., 2014
Mean number of osteocytes in contact with single osteoblast	$\langle \tilde{k}\rangle \text{Ob}=1$ (Appendix E.6)	No units	Kamioka et al., 2001
Osteoblast surface density	$\tilde{p}=6\times 10^{3}$	mm^−2^	Buenzli et al., 2014
Osteocyte density	$\tilde{q}=2.375\times 10^{4}$	mm^−3^	Buenzli and Sims, 2015
**Model dependent parameters (see section** **2.1)**.			
Dendrite growth scale parameter	α (model dependent)	day^−1^	−
Network-independent osteoblast differentiation scale parameter	λ (model dependent)	day^−1^	−
Network-dependent osteoblast differentiation scale parameter	γ (model dependent)	day^−1^	−

One aspect ignored in our model is mechanotransduction. It is known that mechanical loads and fluid flow sheer stress can lead to greater dendrite growth (Zhang et al., 2006; Burra et al., 2010). After now posing our model, a pertinent question then remains as to the relative effect sizes between mechanical stimuli and microenvironmental signaling.

#### 3.2.2. Osteocyte Degree Distribution

In the steady-state traveling wave regime, one can show that the node degrees of the osteocyte network are geometrically distributed when using either the null model (see section 2.1.1), or the switch-like proposed mechanism (see section 2.1.3). This effect comes from the difference equation structure shown in (23)–(25) in Appendix C.1.

In Kerschnitzki et al. (2013), a three-dimensional osteocyte network was studied, the topology of this network includes dendrites that do not connect to a second osteocyte, and connections that link between multiple osteocytes. Additionally, the nodes of their network included both osteocytes and the branching points of dendrites. Thus the functional communication network we studied is different from the lacuno-canalicular pore network and does not account for all types of communication redundancies that may exist. To incorporate all possible redundancies would require the inclusion of connections that do not connect to other nodes, and connections that exist between multiple nodes. We have some redundancy in our communication network in the form of multiconnections (multiple connections between two nodes). However in the limit of large networks, the probability of a multiconnection occurring in our model approaches zero. Even in the case of finite networks, this is very unlikely to occur in the model as $\langle \tilde{k}\rangle \text{Ob},\langle \tilde{k}\rangle \text{Ot}=O\left(1\right)$. Regardless of these model technicalities, it should be noted that the degree distribution of the three-dimensional scanned network in Kerschnitzki et al. (2013) was also shown to be geometrically distributed as derived from our model.

#### 3.2.3. Orientation of Dendrites

It is clear from Figure 3 that we observe orientation of connections between older osteocytes and younger osteocytes or osteoblasts. This leads to an interesting question is as to whether one can configure our model to modify this orientation.

In brief, our model has allowed us to mechanistically explain osteocyte networks in the bone during bone remodeling and explain how these networks are impacted by cancer. Our approach identified, using experimental data, the parameters that characterize find new biology and derive new parameters A possible future direction in our work may be to modify our model to explore the functional difference between lateral connections, and connections perpendicular to the bone surface. As osteocytes have coordinated deposition, one may be able to explore whether coordinated terminal differentiation can occur as a function of lateral connections, burying a group of osteoblasts simultaneously.

## Data Availability Statement

All datasets generated for this study are included in the article/Supplementary Material.

## Author Contributions

JT-K, PB, SC, and DB designed the research. JT-K produced the mathematical model under supervision by PB, SC, and DB. Biological analysis of bone sections was performed by JT-K and CL. The manuscript was written by all authors.

## Conflict of Interest

The authors declare that the research was conducted in the absence of any commercial or financial relationships that could be construed as a potential conflict of interest.

## Footnotes

^1^These include Receptor Activator of Nuclear Factor Kappa-B Ligand (RANKL), Vascular Endothelial Growth Factor (VEGF) (Jilka et al., 2013), Parathyroid Hormone (PTH) (Xiong et al., 2014), calcium ions (Ca^2+^) (Ishihara et al., 2012), and Sclerostin (Sapir-Koren and Livshits, 2014).
