# Supplementary material for: Modeling Osteocyte Network Formation: Healthy and Cancerous Environments
Source: Front Bioeng Biotechnol. 2020 Jul 22;8:757. doi: 10.3389/fbioe.2020.00757 (PMC7387425; doi:10.3389/fbioe.2020.00757)
Supplement: Supplementary file 1 [file Data_Sheet_1.pdf]

Modelling Osteocyte Network Formation: Healthy and Cancerous Environments

Jake P. Taylor-King[1,2,3,*], Pascal R. Buenzli[4], S. Jon Chapman[2], Conor C. Lynch[5], and David Basanta[3,*].

1 Institute of Molecular Systems Biology, Department of Biology, ETHZ, CH-8093 Zurich, Switzerland
2 Mathematical Institute, University of Oxford, Oxford, OX2 6GG, UK
3 Integrated Mathematical Oncology, H. Lee Moffitt Cancer Center and Research Institute, Tampa, FL, USA
4 School of Mathematical Sciences, Queensland University of Technology, Brisbane, QLD 4001, Australia
5 Department of Tumor Biology, H. Lee Moffitt Cancer Center and Research Institute, Tampa, FL, USA

* jake@juvenescence.ltd, david@CancerEvo.org

1 Image processing technique for Osteocyte density calculation

Using the inbuilt functions of the MATLAB image processing toolbox, our technique for calculating approximate osteocyte densities consists of three stages:

1. Colour-based segmentation to isolate the mineralised section of the bone.
2. Entropy-based segmentation to isolate and locate osteocytes centers.
3. Estimation of osteocyte density as

$$\text{Implied osteocyte density} = \frac{\#\text{osteocytes}}{\text{mineralised area}} \tag{1}$$

Colour-based segmentation is carried out by treating the RGB signal as a vector and clustering these vectors using k-means clustering, the value of k chosen depending on an *ad-hoc* basis depending on the specific image. Entropy-based segmentation and thresholding was used to detect approximate locations of osteocytes. Manual validation was also carried out to confirm/correct the locations of the osteocytes detected. In Figure 1, we see the output of the algorithm, the stroma appears red, the mineralised region of the bone appears blue. The region between measured is outlined in green and the osteocyte's center is marked with a red circle.

Our criterion for what was marked and counted as an osteocyte was as follows:

i.) The cell boundary of the osteocyte must be visible.

ii.) There must be de-calcified osteoid around the osteocyte.

2 Monte Carlo Algorithm for Stochastic Simulation of Bone Formation

We give pseudocode for a fixed time step Monte Carlo algorithm for our osteocyte network formation model described in Section **??** using functions and parameters as detailed in Table **??** and mathematical details in

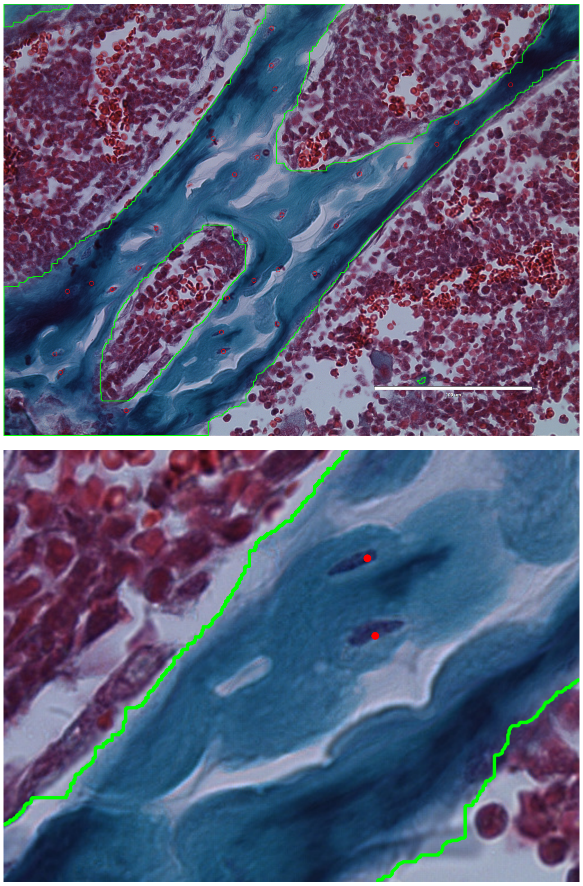

Fig 1. Output of image processing technique for osteocyte density calculation: (top) full size image, (bottom) close-up image. Region measured is outlined in green and osteocyte centers are marked in red.

Table 1. The Monte Carlo algorithm is broken town into two sub-algorithms. Algorithm 1 is the master algorithm that calls on Algorithms 2 and 3.

Algorithm 2 details for how the network structure changes as the following Poisson processes: connection creation events from osteocyte to osteoblast; osteoblast-to-osteocyte differentiation; and osteoblast proliferation. Algorithm 2 follows from the Algorithm given in the appendix of [**?**].

Algorithm 3 details the changing domain $\Omega(t)$ and boundary $B(t)$ (we specify osteoblast migration as part of this). For the changing domain, one has a few options regarding how one deals with the boundary (e.g., the level set method), but due to the fact that we have osteoblasts that occupy positions on the boundary, we use a particle method (discretising the boundary with an ordered set of particles).

As osteoblasts can diffuse on the boundary $B(t)$, osteoblast diffusion is incorporated into this Algorithm 3 via a position jump process (a type of random walk) where osteoblasts can jump either left or right along the discretised boundary as a Poisson process that approximates diffusion (Brownian motion) as the spacing between the boundary discretisation reduces to zero. Therefore, osteoblasts move perpendicular to $B(t)$, and secrete bone normal to $\Omega(t)$.

We now give extra details for Algorithm 3 on the particle method for the changing boundary and the selection of the jumping rate for the osteoblasts.

Algorithm 1: Master algorithm for fixed time step progression of $\Delta t > 0$ that refers to Algorithms 2 and 3.

Data: Choose an end time $T_{\text{end}} = M\Delta t$ for large $M \in \mathbb{N}$ and small $\Delta t > 0$.
Set the number of particles $N \leftarrow N_0$.
Initialize the starting state as $\boldsymbol{s}_i \leftarrow \boldsymbol{s}_0^{(i)} = (\boldsymbol{x}_0^{(i)}, \rho_0^{(i)})$ for $i = 1, \ldots, N$ and $\rho_0 \in \{\text{Ob}, \text{Ot}\}$, and specify starting degree $k_i \leftarrow k_0^{(i)}$.
Specify region $\Omega \leftarrow \Omega_0$ and boundary $B \leftarrow B_0$. (Note that a method for the discretisation of the boundary will be needed.)
Set time counter $m \leftarrow 0$.
while $m \leq M$ **do**
    % Network structure update.
    Run Algorithm 2.
    % Domain size update.
    Run Algorithm 3.
    % Time update.
    Update time: $m \leftarrow m + 1$.

2.1 Extra details for Algorithm 3

Algorithm 3 deals with the changing boundary by allowing for the addition and removal of extra discretisation points. When pairs of discretisation points are too close, one of the points may be removed to avoid having to reduce the time-step Δt. When pairs of discretisation points move to far apart, a new discretisation point is added with coordinates equal to the mean of the pair of discretisation points in question.

There is the potential for topology changes where osteoblasts would be buried without differentiation. However, we do not account for topology changes as circular regions with an osteocyte within them would shrink to a point; we save on computation time by shrinking closed to loops to points immediately (see Figure 2).

Algorithm 3 approximates osteoblast diffusion via a jumping process along the boundary discretisation. The effective jump rates are found from analysing the Taylor expansion for the Poisson process where one either jumps "up" a distance of ℓ_u at rate j_u or "down" a distance of ℓ_d at rate j_d along an arc length coordinate s. As an approximation, we assume that the migration speed of osteoblasts occurs at an order of magnitude faster than the rate of osteoid deposition; therefore we can assume the interface is temporarily static. Consider a small time step of size $\Delta t > 0$, and denote the density of osteoblasts on B by $\rho(t, s)$, then

$$\begin{aligned}\rho(t + \Delta t, s) = \rho(t, s) + \Delta t(j_d \rho(t, s + \ell_d) + j_u \rho(t, s - \ell_u)) \\ - \Delta t(j_d + j_u)\rho(t, s) + \mathcal{O}(\Delta t^2)\,. \end{aligned} \tag{2}$$

Taylor expanding around the value s, and taking the limit as $\Delta t \to 0$, we obtain the drift-diffusion equation

$$\frac{\partial \rho}{\partial t} = (j_d \ell_d - j_u \ell_u)\frac{\partial \rho}{\partial s} + \frac{j_d \ell_d^2 + j_u \ell_u^2}{2}\frac{\partial^2 \rho}{\partial s^2} + \mathcal{O}(\ell_d^3, \ell_u^3)\,. \tag{3}$$

To prevent artificial drift of osteoblasts along the bone surface, one must choose $j_d \ell_d = j_u \ell_u$ and we specify that the diffusion constant is $\kappa_{\text{diff}} = j_d \ell_d^2 + j_u \ell_u^2/2$. Therefore we select the jumping rates j_u and j_d as

$$j_u = \frac{2\kappa_{\text{diff}}}{\ell_u(\ell_d + \ell_u)}\,, \text{ and } j_d = \frac{2\kappa_{\text{diff}}}{\ell_d(\ell_d + \ell_u)}\,. \tag{4}$$

Algorithm 2: Algorithm for network structure changes. By $\texttt{randperm}(X)$, we mean a permutation of the discrete set X selected uniformly at random.

```
% Edge creation update.
for i ← randperm({1, 2, ..., N}) and ϱ_i = Ot do
    for j ← randperm({i + 1, ..., N}) and ϱ_j = Ob do
        Draw a uniform random number r_1 from distribution R ~ U(0, 1).
        if r_1 ≤ C(x_i, x_j)Δt then
            Create an connection between osteocyte i and osteoblast j.

% Differentiation update.
for i ← randperm({1, 2, ..., N}) and ϱ_i = Ob do
    Draw a uniform random number r_2 from distribution R ~ U(0, 1).
    if r_2 ≤ D_k Δt then
        Osteoblast i differentiates into an osteocyte.

% Proliferation update.
for i ← randperm({1, 2, ..., N}), ϱ_i = Ob and k_i = 0 do
    Draw a uniform random number r_3 from distribution R ~ U(0, 1).
    if r_3 ≤ μ(mΔt)Δt then
        Osteoblast i produces a daughter osteoblast. The daughter osteoblast will have index N + 1.
        Initialise position x_{N+1} ← x_i.
        Initialise degree k_{N+1} ← 0.
        Update number of particles: N ← N + 1.
```

3 Mathematical description

We make the model description more precise through the introduction of mathematical concepts and notation. In our model there are two species: osteocytes and osteoblasts. These species act as nodes in a spatial network. Between pairs of nodes, undirected unweighted connections represent dendrites growing away from osteocytes. The osteocytes occupy an expanding domain $\Omega(t) \subset \mathbb{R}^d$, and osteoblasts are located on sections of the boundary of the domain $B(t) \subset \partial\Omega(t)$. The boundary $B(t)$ moves in the outward normal direction $\boldsymbol{n}(t, \boldsymbol{x})$ for $\boldsymbol{x} \in B(t)$ at velocity $\boldsymbol{u}(t, \boldsymbol{x})$. In Fig. 3, we give a schematic of a general domain.

For all times $t > 0$, the whole system is described completely by 4 (multidimensional) quantities. First, one must know how many nodes n are present. Second, we specify the cell type ϱ_i for each node $i = 1, \dots, n$. We must know whether it is an osteoblast (Ob) or an osteocyte (Ot). Third, each node $i = 1, \dots, n$ must have an associated position, we write $\boldsymbol{x}_i = (x_1, \dots, x_d) \in \Omega(t) \cup B(t) \subset \mathbb{R}^d$. Finally, we must know the adjacency matrix of the underlying network. The adjacency matrix of a network is a matrix $A_n \in \mathbb{N}_0^{n\times n}$ with entries that detail the number of unweighted undirected connections between nodes i and j. Our framework [**?**] allows for the existence of multiconnections (multiple connections between the same two nodes) for mathematical tractability. Although as the size of the network becomes large, the probability of multiconnections occurring approaches zero. Additionally, the mean degree for each node is *a priori* likely to be low, e.g., we used $\langle \tilde{k} \rangle_{\mathrm{Ob}} = 1$.

We now respecify the model given in Figure **??** (and mathematical in Table 1). We frequently make use of the phrasing that *events occur at some rate*. This rate will always then refer to the probability of an event happening per unit time (also known as a Poisson process).

We list the functional forms underpinning our model in Table **??**; in the case of the rate of osteoblast differentiation (D_k), we investigate possible choices that represent different dependences on network

structure. Recurring parameters that have fixed values used are listed in Table ??. Both Tables ?? and ?? contain brief details on how function forms/parameters were chosen; for extra details, see Appendix 5.

3.1 Derivation of mean-field equations

From earlier work [?], we can write down differential equations for the Local State Degree Distributions (LSDD). In Ref. [?], the expression $u_k(t, \boldsymbol{s})$ was the expected number of particles with degree k and state vector $\boldsymbol{s}$. For the model contained in Section 3, our state vector is described by position and cell type, so $\boldsymbol{s} = (\boldsymbol{x}, \varrho)$, and $\varrho \in \{\mathrm{Ob}, \mathrm{Ot}\}$.

For notational convenience, we write $v_k(t, \boldsymbol{x})$ is the expected number of osteoblasts of degree k with position $\boldsymbol{x}$ at time t, and $w_k(t, \boldsymbol{x})$ as the expected number of osteocytes of degree k with position $\boldsymbol{x}$ at time t. We write the number density of osteoblasts and osteocytes (respectively) as

$$p(t, \boldsymbol{x}) = \sum_{k=0}^{\infty} v_k(t, \boldsymbol{x}) \, , \quad q(t, \boldsymbol{x}) = \sum_{k=0}^{\infty} w_k(t, \boldsymbol{x}) \, . \tag{9}$$

The osteocytes are present in domain $\Omega(t) \subset \mathbb{R}^d$, and the osteoblasts are present on (a subset of) the boundary of this domain $B(t) \subset \partial\Omega(t)$. The domain boundary moves with normal velocity

$$\boldsymbol{u}(t, \boldsymbol{x}) = \nu(t, \boldsymbol{x}) \boldsymbol{n}(t, \boldsymbol{x}) \, , \tag{10}$$

where $\boldsymbol{n}$ is the normal to $\partial\Omega$. Using Stone's derivation of surfactants on moving interfaces [?], our equations become

$$\begin{aligned} &\frac{\partial v_k(t, \boldsymbol{x})}{\partial t} + \nabla_S \cdot (\boldsymbol{u}(t, \boldsymbol{x}) v_k(t, \boldsymbol{x})) = -D_k v_k(t, \boldsymbol{x}) \\ &+ \left(\int_{\Omega(t)} \mathcal{C}(\boldsymbol{x}, \boldsymbol{y}) q(t, \boldsymbol{y}) \mathrm{d}\boldsymbol{y} \right) [v_{k-1}(t, \boldsymbol{x}) - v_k(t, \boldsymbol{x})] \\ &+ \delta_{k,0} \left(\kappa_{\mathrm{diff}} \nabla_S^2 v_0(t, \boldsymbol{x}) + \mu(t) v_0(t, \boldsymbol{x}) \right) \, , \end{aligned} \tag{11}$$

in $B(t)$, and

$$\begin{aligned} &\frac{\partial w_k(t, \boldsymbol{x})}{\partial t} = D_k v_k(t, \boldsymbol{x}) \delta_{B(t)}(\boldsymbol{x}) \\ &+ \left(\int_{B(t)} \mathcal{C}(\boldsymbol{x}, \boldsymbol{y}) p(t, \boldsymbol{y}) \mathrm{d}\boldsymbol{y} \right) [w_{k-1}(t, \boldsymbol{x}) - w_k(t, \boldsymbol{x})] \, , \end{aligned} \tag{12}$$

in $\Omega(t)$, where $\nabla_S := (I_d - \boldsymbol{n}\boldsymbol{n}^T)\nabla$ is the surface gradient operator[1];

Taking the continuum limit in equation (5), then

$$\nu(t, \boldsymbol{x}) = \int_{B(t)} s(\boldsymbol{x}, \boldsymbol{y}) p(t, \boldsymbol{y}) \mathrm{d}\boldsymbol{y} \, . \tag{13}$$

Assuming a solution homogenous in the y and z directions, we can write the system's domain as: $\Omega(t) = (-\infty, \omega(t)]$; the normal vector as $\boldsymbol{n} = \boldsymbol{e}_x$; and then

$$v_k(t, \boldsymbol{x}) \equiv v_k(t, \omega(t)) \equiv v_k(t) \, , \quad w_k(t, \boldsymbol{x}) \equiv w_k(t, x) \, . \tag{14}$$

[1] We use standard convention that I_d is the d-dimensional identity matrix.

Under these assumptions, equations (11) and (12) become

$$\frac{\mathrm{d}v_k(t)}{\mathrm{d}t} = -D_k v_k(t) + \delta_{k,0}\mu(t)v_0(t) + \left(\int_{-\infty}^{\omega(t)} K(\omega(t), y)\, q(t,y)\mathrm{d}y\right)[v_{k-1}(t) - v_k(t)]\,, \tag{15}$$

on the bone surface $B(t) = \{\omega(t)\}$ for kernel $K(x,y) = \alpha\beta L_z\sqrt{2\pi}\exp\left\{-\frac{1}{2}\beta^{-2}|x-y|^2\right\}$, and

$$\frac{\partial w_k(t,x)}{\partial t} = D_k v_k(t)\delta(\omega(t) - x) + K(\omega(t), x)\, p(t)\,[w_{k-1}(t,x) - w_k(t,x)]\,, \tag{16}$$

in the bone volume $\Omega(t)$. The velocity given by equation (13) is then interpreted as an ODE for the position of $\omega(t)$, which is

$$\frac{\mathrm{d}\omega(t)}{\mathrm{d}t} = \kappa_{\mathrm{form}} p(t) = \nu(t)\,, \tag{17}$$

for secretion rate

$$\kappa_{\mathrm{form}} = \eta\iota\sqrt{2\pi}L_z\,, \tag{18}$$

which represents the volume of bone secreted per osteoblast per unit time.

Through the use of the method of characteristics, we can write equation (16) as an integral of the solution of equation (15), see Appendix 4.1 for full details. One can then use a standard finite differences numerical scheme to solve equation (15).

4 Solution method

We now need to solve equations (15), (16), and (17). We consider two different scenarios. In the first case (Section 4.1), we turn the differential equations for $w_k(t,x)$ into integral equations involving the solution to $v_k(t)$ so that we only need to construct a numerical scheme for the ODEs for $v_k(t)$. In the second case (Section 4.2), we choose the rate of proliferation μ to conserve mass [See equation (40)], allowing a travelling wave solution which can be solved analytically.

4.1 Time dependent problem.

Analysing equations with a moving boundary can be challenging, so we change our coordinate system to a fix the location of this boundary by moving from (x,t) to (z,τ), where $z = x - \omega(t)$ and $\tau = t$. Therefore we write equation (16) as

$$\frac{\partial w_k(\tau,z)}{\partial \tau} - \omega'(\tau)\frac{\partial w_k(\tau,z)}{\partial z} \tag{19}$$

$$= K(\omega(\tau), \omega(\tau) + z)\, p(\tau)\,[w_{k-1}(\tau,z) - w_k(\tau,z)]\,, \tag{20}$$

on $(z,\tau) \in \mathbb{R}_- \times \mathbb{R}_+$ with initial condition

$$w_k(\tau = 0, z) = f_k^{(0)}(z)\,. \tag{21}$$

Additionally, we turn the delta function in equation (16) into the boundary condition

$$w_k(\tau, z=0) = \frac{D_k v_k(\tau)}{\omega'(\tau)} = g_k^{(0)}(\tau)\,. \tag{22}$$

We use the Method of Characteristics using ζ as the characteristic variable, we find

$$\frac{\mathrm{d}\tau}{\mathrm{d}\zeta} = 1\,, \tag{23}$$

$$\frac{\mathrm{d}z}{\mathrm{d}\zeta} = -\omega'(\tau)\,, \tag{24}$$

$$\frac{\mathrm{d}w_k}{\mathrm{d}\zeta} = K(\omega(\tau), \omega(\tau)+z)\,p(\tau)\,[w_{k-1} - w_k]\,. \tag{25}$$

When $\zeta = 0$, we specify initial and boundary data as a function of ξ. We write

$$\tau = \tau_0(\xi) = \begin{cases} \xi & \text{if} \quad \xi > 0\,, \\ 0 & \text{if} \quad \xi < 0\,, \end{cases} \tag{26}$$

$$z = z_0(\xi) = \begin{cases} 0 & \text{if} \quad \xi > 0\,, \\ \xi & \text{if} \quad \xi < 0\,, \end{cases} \tag{27}$$

$$w = w_k(\xi) = \begin{cases} g_k^{(0)}(\xi) & \text{if} \quad \xi > 0\,, \\ f_k^{(0)}(\xi) & \text{if} \quad \xi < 0\,. \end{cases} \tag{28}$$

Solving equations (23) and (24), we find that

$$\tau = \begin{cases} \zeta + \xi & \text{if} \quad \xi > 0\,, \\ \zeta & \text{if} \quad \xi < 0\,, \end{cases} \tag{29}$$

$$z = \begin{cases} \omega(\xi) - \omega(\zeta+\xi) & \text{if} \quad \xi > 0\,, \\ \omega_0 - \omega(\zeta) + \xi & \text{if} \quad \xi < 0\,. \end{cases} \tag{30}$$

Writing

$$\epsilon(\zeta, \xi) = \begin{cases} \int_0^\zeta K(\omega(y+\xi), \omega(\xi)) p(y+\xi) \mathrm{d}y & \text{if} \quad \xi > 0\,, \\ \int_0^\zeta K(\omega(y), \omega_0 + \xi) p(y) \mathrm{d}y & \text{if} \quad \xi < 0\,, \end{cases} \tag{31}$$

then

$$w_k(\zeta, \xi) = e^{-\epsilon(\zeta,\xi)} \sum_{j=0}^{k} w_{k-j}(\tau = 0, \xi) \frac{\epsilon(\zeta,\xi)^j}{j!}\,. \tag{32}$$

Inverting equations (29) and (30), we obtain

$$\zeta = \begin{cases} \tau - \omega^{-1}[\omega(\tau) + z] & \text{if} \quad z > \omega_0 - \omega(\tau)\,, \\ \tau & \text{if} \quad z < \omega_0 - \omega(\tau)\,, \end{cases} \tag{33}$$

$$\xi = \begin{cases} \omega^{-1}[\omega(\tau) + z] & \text{if} \quad z > \omega_0 - \omega(\tau)\,, \\ z + \omega(\tau) - \omega_0 & \text{if} \quad z < \omega_0 - \omega(\tau)\,, \end{cases} \tag{34}$$

$$\tag{35}$$

and therefore the solution as a function of (z, τ) is given by inserting equations (33) and (34) into equation (32). For the exponent term $\epsilon(z, \tau)$, we can use a change of variables and write

$$\epsilon(z, \tau) = \begin{cases} \int_{\omega^{-1}[\omega(\tau)+z]}^{\tau} K(\omega(h), \omega(\tau)+z) p(h) \mathrm{d}h & \text{if} \quad z > \omega_0 - \omega(\tau)\,, \\ \int_0^\tau K(\omega(h), \omega(\tau)+z) p(h) \mathrm{d}h & \text{if} \quad z < \omega_0 - \omega(\tau)\,. \end{cases} \tag{36}$$

and we have also therefore solved our equations for $w_k(t,x)$. To solve equation (15) for $v_k(t)$, we need to evaluate the integral

$$F(t) := \int_{-\infty}^{\omega(t)} K(\omega(t),y)\, q(t,y)\mathrm{d}y\,. \tag{37}$$

By noting that

$$q(t,x) = \sum_{k=0}^{\infty} \begin{cases} g_k^{(0)}(\omega^{-1}(x)) & \text{if} \quad x > \omega_0\,, \\ f_k^{(0)}(x-\omega_0) & \text{if} \quad x < \omega_0\,, \end{cases} \tag{38}$$

then we can calculate F as the contribution from the initial condition $f_k^{(0)}$ and the boundary condition $g_k^{(0)}$, therefore

$$\begin{aligned} F(t) = & \sum_{k=0}^{\infty} \int_{-\infty}^{\omega_0} K(\omega(t),x) f_k^{(0)}(x-\omega_0)\mathrm{d}x \\ & + \sum_{k=0}^{\infty} \int_{\omega_0}^{\omega(t)} K(\omega(t),x) g_k^{(0)}(\omega^{-1}(x))\mathrm{d}x\,. \end{aligned} \tag{39}$$

Referring back to the definition of $g_k^{(0)}$ in equation (22), we note that our ODEs have become integro-differential equations. These can be solved via finite differences.

4.2 Time independent problem: mass conserving travelling waves.

To maintain a constant surface density of osteoblasts, we must choose

$$\mu = \mu(\{v_k(t)\}_{k=0}^{\infty}) = \frac{\sum_{k=0}^{\infty} D_k v_k(t)}{v_0(t)}\,, \tag{40}$$

so that

$$\frac{\mathrm{d}p(t)}{\mathrm{d}t} = \sum_{k=0}^{\infty} \frac{\mathrm{d}v_k(t)}{\mathrm{d}t} = 0\,. \tag{41}$$

Our system of ODEs then admits a travelling wave solution corresponding uniform bone growth. We write the travelling wave speed as $\tilde{\nu}$, the solutions of the ODEs now correspond to the constant value $\tilde{v}_k$ with sum $\tilde{p} = \sum_k \tilde{v}_k$. The solutions for the degree k osteocytes have travelling wave profile $\tilde{w}_k(x)$ where $x \to -\infty$ corresponds to the back of the wave (away from the osteoblast front) and $x = \omega_0$ is the front of the wave (where the osteoblasts are located). The total osteocyte density $\tilde{q} = \sum_k \tilde{w}_k(x)$ is then a constant by equation (38) as the initial condition $f_k^{(0)}$ does not enter into the solution.

From equation (17), the wave speed is given by the relation

$$\tilde{\nu} = \eta \iota L_z \sqrt{2\pi}\tilde{p}\,. \tag{42}$$

We now have to solve the algebraic system of equations

$$\begin{aligned} -D_k\tilde{v}_k + \delta_{k,0} \sum_{l=0}^{\infty} D_l^{(\mathrm{b})}\tilde{v}_l & \\ + \tilde{F}[\tilde{v}_{k-1} - \tilde{v}_k] = 0\,. & \end{aligned} \tag{43}$$

The constant $\tilde{F}$ from equation (37) from is given by

$$\begin{aligned}\tilde{F} &= \left[\int_{-\infty}^{\omega_0} K(\omega_0, x)\mathrm{d}x\right]\tilde{q} \\ &= \left[\frac{1}{2}\int_{-\infty}^{\infty} \alpha\beta L_z\sqrt{2\pi}\exp\{-\frac{x^2}{2\beta^2}\}\mathrm{d}x\right]\tilde{q} \\ &= \alpha\beta^2\pi L_z\tilde{q}\,. \end{aligned} \tag{44}$$

Summing equation (22) over k and using equation (42), $\tilde{q}$ is given by

$$\tilde{q} = \frac{\sum_{k=0}^{\infty} D_k\tilde{v}_k}{\eta\iota\sqrt{2\pi}L_z\tilde{p}}\,. \tag{45}$$

The mean osteoblast degree can be found by solving equations (43) and calculating $\langle\tilde{k}\rangle_{\mathrm{Ob}} = \sum_k k\tilde{v}_k/\tilde{p}$. We can also calculate the solution to $\tilde{w}_k(x)$ by using equation (32) and assuming steady-state bone formation, that is

$$\tilde{w}_k(x) = e^{-\tilde{\epsilon}(x)}\sum_{j=0}^{k}\tilde{g}_{k-j}\frac{\tilde{\epsilon}(x)^j}{j!}\,, \tag{46}$$

where

$$\tilde{g}_k = \frac{D_k\tilde{v}_k}{\tilde{\nu}}\,, \tag{47}$$

and

$$\tilde{\epsilon}(x) = \frac{\tilde{p}}{\tilde{\nu}}\int_x^{\omega_0} K(y, x)\mathrm{d}y\,. \tag{48}$$

With a solution to $\{\tilde{v}_k\}_{k=0}^{\infty}$, then the mean osteocyte degree of connectivity can be calculated away from the osteoblast front as

$$\begin{aligned}\langle\tilde{k}\rangle_{\mathrm{Ot}} &= \lim_{x\to-\infty}\frac{\sum_{k=0}^{\infty} k\tilde{w}_k(x)}{\tilde{q}} \\ &= \lim_{x\to-\infty}\frac{e^{-\tilde{\epsilon}(x)}\sum_{k=0}^{\infty} k\,\sum_{j=0}^{k}\tilde{g}_{k-j}\frac{\tilde{\epsilon}(x)^j}{j!}}{\tilde{q}}\,, \\ &= \lim_{x\to-\infty}\frac{e^{-\tilde{\epsilon}(x)}\sum_{k=0}^{\infty}\tilde{g}_k\sum_{j=0}^{\infty}(k+j)\frac{\tilde{\epsilon}(x)^j}{j!}}{\tilde{q}}\,, \\ &= \lim_{x\to-\infty}\frac{\sum_{k=0}^{\infty} k\,\tilde{g}_k + \tilde{q}\,\tilde{\epsilon}(x)}{\tilde{q}}\,, \\ &= \frac{\sum_{k=0}^{\infty} kD_k\tilde{v}_k}{\sum_{k=0}^{\infty} D_k\tilde{v}_k} + \frac{\alpha\beta^2}{\eta\iota}\sqrt{\frac{\pi}{2}}\,. \end{aligned} \tag{49}$$

We then use equation (49) for different choices in D_k to aid with parameterisation.

4.2.1 No network influence: Null hypothesis

Rewriting equation (**??**) below

$$D_k^{(\mathrm{null})} = \hat{D}\,,$$

one can insert this choice of D_k into equations (43)–(45) to show that the osteocyte number density is

$$\tilde{q} = \frac{\hat{D}}{\eta\iota\sqrt{2\pi}L_z}\,, \tag{50}$$

the mean osteoblast degree is

$$\langle \tilde{k} \rangle_{\text{Ob}} = \frac{\hat{\alpha}\beta^2}{\eta\iota}\sqrt{\frac{\pi}{2}}\,, \tag{51}$$

and the mean osteocyte degree of connectivity is

$$\langle \tilde{k} \rangle_{\text{Ot}} = \frac{\hat{\alpha}\beta^2}{\eta\iota}\sqrt{2\pi}\,. \tag{52}$$

Quite remarkably, it is always the case that $\langle \tilde{k} \rangle_{\text{Ot}} = 2\langle \tilde{k} \rangle_{\text{Ob}}$.

4.2.2 Proposed mechanism: Switch-like influence

Rewriting equation (**??**),

$$D_k^{(\text{swt})} = \begin{cases} \lambda_{\text{swt}} & \text{if} \quad k = 0\,, \\ \lambda_{\text{swt}} + \gamma_{\text{swt}} & \text{if} \quad k \geq 1\,, \end{cases}$$

one can insert this choice of D_k into equations (43)–(45) to show that

$$A\tilde{q} = (\lambda_{\text{swt}} + \gamma_{\text{swt}})\langle \tilde{k} \rangle_{\text{Ob}}\,, \tag{53}$$

for $A = \alpha_{\text{swt}}\beta^2\pi L_z$ and

$$\langle \tilde{k} \rangle_{\text{Ob}}^2 + \left(1 - \frac{A}{B}\right)\langle \tilde{k} \rangle_{\text{Ob}} - \frac{A}{B}\frac{\lambda_{\text{swt}}}{\lambda_{\text{swt}} + \gamma_{\text{swt}}} = 0\,, \tag{54}$$

for $B = \eta\iota\sqrt{2\pi}L_z$. The solutions of the equations above are algebraically tumultuous — so we do not present these here! Additionally, the mean osteocyte degree of connectivity is

$$\langle \tilde{k} \rangle_{\text{Ot}} = \frac{\tilde{F}(\lambda_{\text{swt}} + \gamma_{\text{swt}} + \tilde{F})}{(\lambda_{\text{swt}} + \gamma_{\text{swt}})(\lambda_{\text{swt}} + \tilde{F})} + \frac{\alpha\beta^2}{\eta\iota}\sqrt{\frac{\pi}{2}}\,. \tag{55}$$

Note there is consistency that the solutions to equations (53)–(55) agree with the solutions to equations (50)–(52) when $\gamma_{\text{swt}} = 0$.

5 Parameter estimation

5.1 Osteoblast diffusion constant ($\kappa_{\textbf{diff}}$)

In the work by Araujo *et. al.* [**?**], a cellular automata model was proposed in which migrating osteoblasts were allowed to move on a lattice every time step. With time steps of 6 mins, in the absence of any chemical signalling, osteoblasts moved either up, down, left or right with equal probability. Additionally, it was stated that (from experiments), the speed of a migrating osteoblast was 0.1470 mm day^{-1}.

Using the results from Taylor-King *et. al*, it was reported for a velocity jump process in n-dimensions with fixed speed S_T, exponentially distributed runs of mean length μ_τ, and with uniformly random selection of new directions, the large time effective diffusion constant is

$$\kappa_{\text{diff}} = \frac{S_T^2\mu}{n} = \frac{(0.1470)^2(\frac{6}{60}\frac{1}{24})}{2} = 4.50 \times 10^{-5}\,\text{mm}^2\,\text{day}^{-1}\,. \tag{56}$$

5.2 Domain size (L_z, L_y)

L_y is chosen such that $L_y \gg \beta$. L_z is chosen to be the diameter of an osteoblast (15×10^{-3} mm) plus the mean distance between osteocytes (25×10^{-3} mm). Also note that $\omega_0 = 0$

5.3 Bone secretion rate and shape parameters (η, ι)

Through analysis of the travelling wave case (see Appendix 4.2), one can choose η such that one obtains the (experimentally observed [**?**]) travelling wave speed of $\tilde{\nu} = 0.656\,\mu\mathrm{m\,day}^{-1}$. This is done by noting that for a constant wave speed $\tilde{\nu}$, by equation (17), we choose that

$$\eta = \frac{\tilde{\nu}}{\iota L_z \tilde{p}\sqrt{2\pi}}\,. \tag{57}$$

The shape parameter ι is essentially the standard deviation of the normally distributed bone secretion function and is chosen to be the typical diameter of an osteoblast.

5.4 Dendrite growth shape parameter (β)

This value is essentially the standard deviation of the normally distributed rate function [Equation (7)]. Note that we are not selecting the length of dendrites with this parameter, it specifies a how likely a dendrite is likely to form from an osteocyte within the bone to an osteoblast on the bone surface. The length of the dendrite may change while the osteoblast is located on the bone surface, once it differentiates into an osteocyte, the connection length is fixed.

We choose β to be the typical distance between osteocytes. The typical distance measured between osteocytes was reported in Ref. [**?**] to be between 10–40 μm; we use the midpoint in this range.

5.5 Osteoblast and osteocyte densities ($\tilde{p}$, $\tilde{q}$)

Osteoblast surface densities are observed within the range 2–10 $\times\ 10^3\,\mathrm{mm}^{-2}$, the value of $\tilde{p}$ was chosen to be the midpoint in this range. Osteocyte densities are observed within the range 1.90–2.85 $\times\ 10^4\,\mathrm{mm}^{-3}$, the value of $\tilde{q}$ was chosen to be the midpoint in this range. Both of these values are used in steady-state travelling wave regime.

5.6 Mean osteoblast degree ($\langle\tilde{k}\rangle_{\mathbf{Ob}}$)

The only network structure property that we deduced from experimental data was the average number of distinct osteocytes that a single osteoblast connects to. This corresponds to the average node degree of osteoblasts in our connectivity network, $\langle\tilde{k}\rangle_{\mathrm{Ob}}$. We assumed this number to be $\langle\tilde{k}\rangle_{\mathrm{Ob}} = 1$ based on the following estimate. We divide the total number of dendritic processes of an osteoblast, by the redundancy of the physiological network, i.e. by the number of dendritic processes connecting the same osteoblast and osteocyte. Kamioka *et. al.* [**?**] measured the number of dendritic processes coming from an osteoblast to be 4.8. The redundancy of osteocyte–osteoblast connections can be estimated by dividing the number of processes running from an osteocyte to osteoblasts (26.05) by the number of osteoblasts reached by a single osteocyte (5.7), giving a redundancy of 4.57 [**?**]. This provides the estimate $\langle\tilde{k}\rangle_{\mathrm{Ob}} \approx 4.8/4.57 \approx 1$. This value is consistent with Kamioka *et. al.*'s observation that they rarely observed osteoblasts that had connections with more than one osteocyte. We note here that there are not many studies reporting these kinds of measurements. Marotti *et. al.* [**?**] have reported the number of dendrites coming from an osteoblast to range from 9.4 to 20.9 in four osteoblasts (so $\sim$ 13.6 on average). If this figure is used instead, $\langle\tilde{k}\rangle_{\mathrm{Ob}} \sim 3$.

6 Discussion on D_k

6.1 Proposed mechanism: Cumulative activation

One mechanism we considered was a cumulative activation effect, essentially each osteoblast has an network-independent rate of differentiation (λ_1) plus an network-dependent contribution ($f = \gamma_1 k$). This network-dependent contribution corresponds to increasing the rate of osteoblast-to-osteocyte differentiation proportional to the number osteocytes that each osteoblast is in contact with. We write

$$D_k^{(1)} = \lambda_1 + \gamma_1 k\,. \tag{58}$$

One can calculate the mean osteoblast degree with the following analysis. Writing the first moment of $\tilde{v}_k$ as $\tilde{\mathcal{M}} = \sum_{k=0}^{\infty} k\tilde{v}_k$, then the equation for $k = 0$ is

$$\lambda_1 \tilde{p} + \gamma_1 \tilde{\mathcal{M}} = (\tilde{F} + \lambda_1)\tilde{v}_0\,. \tag{59}$$

We can recursively find all $\tilde{v}_k$, and we determine that

$$\tilde{v}_k = \tilde{v}_{k-1}\frac{\tilde{F}}{\tilde{F} + \lambda_1 + \gamma_1 k}\,. \tag{60}$$

though the use of lower incomplete gamma functions defined as $\hat{\gamma}(s,x) = \int_0^x t^{s-1}e^{-t}\mathrm{d}t$, one can show that

$$\frac{\hat{\gamma}(s,x)e^x}{x^{s-1}} = \sum_{k=0}^{\infty}\frac{x^k}{(s+1)\ldots(s+k)}\,, \tag{61}$$

and therefore

$$\langle\tilde{k}\rangle_{\mathrm{Ob}} = G\left(\frac{\tilde{F}+\lambda_1}{\gamma_1}, \frac{\tilde{F}}{\gamma_1}\right) - \frac{\lambda_1}{\gamma_1} \tag{62}$$

for $G(s,x) = x^s e^{-x}/\hat{\gamma}(s,x)$ and

$$\tilde{q} = \frac{\lambda_1 + \gamma_1\langle\tilde{k}\rangle_{\mathrm{Ob}}}{\eta\iota\sqrt{2\pi}L_z}\,. \tag{63}$$

We fit parameters as stated in Section **??**. Specifying that the network has an excitatory effect on osteoblast differentiation, we set $\lambda_1 = \hat{D}/2$ and therefore $\alpha_1 = 2.41 \times 10^{-3}\,\mathrm{day}^{-1}$ and $\gamma_1 = 1.30 \times 10^{-3}\,\mathrm{day}^{-1}$. Figure 4 shows that when considering the cumulative activation model when compared to the null model, it takes approximately 3 years to get to the desired osteocyte density of $\tilde{q} = 2.375 \times 10^4\,\mathrm{mm}^{-3}$. This timescale is clearly too long to be considered biologically realistic. Additionally, were one to make $\gamma_1 < 0$, the rate $D_k^{(1)}$ can be negative which means that when using this model, inhibitory network effects are impossible. When considering the cumulative activation model, at the time of osteoblast differentiation a large quantity of a differentiation-promoting protein must be present in the osteoblasts. This protein would have therefore previously diffused along the dendrite structures. From a biological perspective, it seems more likely that only a small amount of protein should be required to travel though the dendrite structure to induce differentiation.

6.2 Proposed mechanism: Diminishing activation

Another mechanism considered is a cumulative inhibition effect. Each osteoblast has an network-independent rate of differentiation (λ_2) plus an network-dependent contribution ($f = \gamma_2/k$). This network-dependent

contribution corresponds to decreasing the rate of osteoblast-to-osteocyte differentiation inversely proportional to the number osteocytes that each osteoblast is contact with. We write

$$D_k^{(2)} = \begin{cases} \lambda_2 & \text{if} \quad k = 0\,, \\ \lambda_2 + \frac{\gamma_2}{k} & \text{if} \quad k \geq 1\,. \end{cases} \,. \tag{64}$$

To calculate the mean osteoblast degree, one can make use of the the series

$$H_f(x, y) = \sum_{k=0}^{\infty} f(k)[y + k + 1]B(y + 1, k + 1)x^k \tag{65}$$

for $f : \mathbb{N}_0 \to \mathbb{R}$ and $B(a, b) = \int_0^1 t^{a-1}(1-t)^{b-1}\mathrm{d}t$ is the beta function to calculate

$$\langle \tilde{k} \rangle_{\mathrm{Ob}} = \frac{H_k(\frac{\tilde{F}}{\tilde{F}+\lambda}, \frac{\gamma}{\tilde{F}+\lambda})}{H_1(\frac{\tilde{F}}{\tilde{F}+\lambda}, \frac{\gamma}{\tilde{F}+\lambda})} \tag{66}$$

and

$$\tilde{q} = \frac{\tilde{F} + \lambda}{\eta\iota\sqrt{2\pi}L_z} \left[H_1\left(\frac{\tilde{F}}{\tilde{F}+\lambda}, \frac{\gamma}{\tilde{F}+\lambda} \right) \right]^{-1} \,. \tag{67}$$

Using equation (44), algebraic equations (66)–(67) are closed.

We fit parameters as stated in Section **??**. Specifying that the network has an excitatory effect on osteoblast differentiation, we set $\lambda_2 = \hat{D}/2$ and therefore $\alpha_2 = 1.75 \times 10^{-3}\,\mathrm{day}^{-1}$ and $\gamma_2 = 4.56 \times 10^{-3}\,\mathrm{day}^{-1}$. Our diminishing activation model also has the possibility to allow for inhibition when $\lambda_2 > \hat{D}$; however the rate $D_k^{(2)}$ can be negative. For the greatest allowable amount of inhibition, one can set $\gamma_2 = -\lambda_2$. To maintain the desired travelling wave profile, the maximum value of λ_2 is given as $\lambda_2 \approx 190\% \times \hat{D}$ and in which case $\alpha_2 = 1.01 \times 10^{-3}\,\mathrm{day}^{-1}$.

Both the excitatory and (maximum) inhibitory effects are shown in Figure 4. When compared to the null model, both models take approximately 2 years to get to the desired osteocyte density. This timescale is too long to be considered biologically realistic.

One emergent property we notice from this choice in differentiation mechanism is that when D_k is non-monotonic (i.e., has a maximum/minimum around $k = 1$), it appears that there is a over-reaching then adjustment period when viewing the density profile $q(t, x)$, e.g., the density starts below $\tilde{q}$, then over-reaches above $\tilde{q}$ before converging to $\tilde{q}$. When viewing 3 dimensional scans, one does observe similar behaviour around cement lines [**? ?**].

Algorithm 3: Algorithm for changing the size of the domain $\Omega(t)$ and boundary $B(t)$ for small time step $\Delta t > 0$.

Data: Specify small time step $\Delta t > 0$ and two tolerances $\mathrm{TOL}_1 > \mathrm{TOL}_2$.

For a boundary discretised by N_B points labelled $\{\boldsymbol{y}_i\}_{i=1}^{N_B}$, the locations of the osteoblasts are constrained to the boundary discretisation $\{\boldsymbol{x}_i\}_{\rho_i=\mathrm{Ob}} \subset \{\boldsymbol{y}_i\}_{i=1}^{N_B}$.

% Osteoblast migration update.

for $i \leftarrow$ `randperm`$(\{1, 2, \dots, N\})$, $\varrho_i = Ob$ *and* $k_i = 0$ **do**

- *% Allow osteoblast i to diffuse on the manifold B.*
- Calculate the rate of jumping up $j_u = 2\kappa_{\mathrm{diff}}/\ell_u(\ell_u + \ell_d)$ and the rate of jumping down as for $j_d = 2\kappa_{\mathrm{diff}}/\ell_d(\ell_u + \ell_d)$ for $\ell_u = ||\boldsymbol{y}_i - \boldsymbol{y}_{i+1}||$ and $\ell_d = ||\boldsymbol{y}_{i-1} - \boldsymbol{y}_i||$.
- Draw a uniform random number r_u from distribution $R \sim \mathcal{U}(0, 1)$.
- **if** $r_u \leq j_u \Delta t$ **then**
  - *% Osteoblast i moves up.*
  - $\boldsymbol{x}_i \leftarrow \boldsymbol{y}_{i-1}$
- Draw a uniform random number r_d from distribution $R \sim \mathcal{U}(0, 1)$.
- **if** $r_d \leq j_d \Delta t$ **then**
  - *% Osteoblast i moves down.*
  - $\boldsymbol{x}_i \leftarrow \boldsymbol{y}_{i+1}$

% Boundary position update; note that the distance between used in $S(\boldsymbol{y}_i, \boldsymbol{x}_j)$ is the curve distance.

for $i \leftarrow \{1, 2, \dots, N_B\}$ **do**

- $\boldsymbol{y}_i \leftarrow \boldsymbol{y}_i + \Delta t\, \boldsymbol{n}(\boldsymbol{y}_i) \sum_{\rho_j=\mathrm{Ob}} S(\boldsymbol{y}_i, \boldsymbol{x}_j)$.

% Boundary node deletion (See Fig. 2a).

for $i \leftarrow$ `randperm`$(\{1, 2, \dots, N_B - 1\})$ **do**

- **if** $||\boldsymbol{y}_i - \boldsymbol{y}_{i+1}|| < TOL_2$ **then**
  - Delete discretisation point $\boldsymbol{y}_i$ provided there is no osteoblast present at this location.

% Boundary node addition (See Fig. 2b).

for $i \leftarrow$ `randperm`$(\{1, 2, \dots, N_B - 1\})$ **do**

- **if** $||\boldsymbol{y}_i - \boldsymbol{y}_{i+1}|| > TOL_1$ **then**
  - Add discretisation point between points $\boldsymbol{y}_i, \boldsymbol{y}_{i+1}$, $\boldsymbol{y}^* = (\boldsymbol{y}_i + \boldsymbol{y}_{i+1})/2$.

% Boundary topology update (See Fig. 2c).

for $i \leftarrow$ `randperm`$(\{1, 2, \dots, N_B - 1\})$ **do**

- **if** $y_{i+1,2} < y_{i,2}$ **then**
  - Change topology by allowing for a closed loop in addition to exterior boundary.
  - *Optional: Save on computation by instantly shrinking loop to single point.*

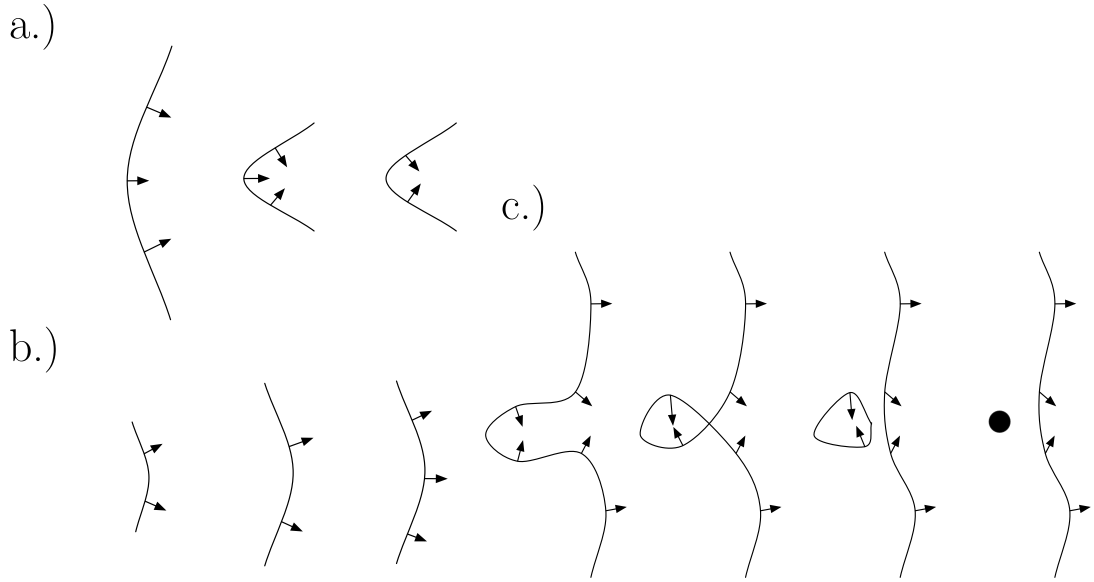

Fig 2. Diagrams showing boundary discretisation refinements: (a) shows a discretisation point being removed as the surrounding points move closer together; (b) shows a discretisation point being added as the surrounding points move further apart; and (c) shows how topology changes may occur, in the far right graphic, a hole is immediately collapsed (which saves on computation time).

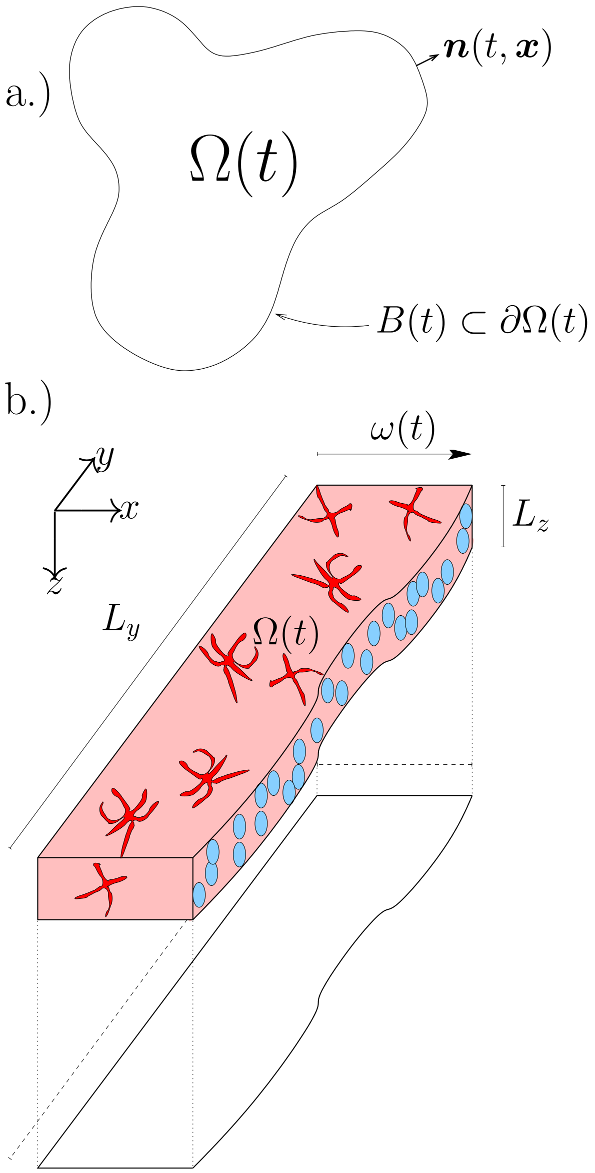

Fig 3. Domain diagrams for mode (a) general diagram of the domain $\Omega(t)$, and (b) domain specific to model realisations. Osteoblasts are depicted in light blue, osteocytes are shown in red. Two dimensional projection shown below.

Table 1. Model description: (i) bone secretion; (ii) osteoblast differentiation; (iii) dendrite growth; (iv) osteoblast migration; and (v) osteoblast proliferation.

(i) The bone surfaces move with normal speed ν depending on the number of nearby osteoblasts. For the diagram shown in Fig. 3, we write

$$\nu(t, \boldsymbol{x}) = \sum_{i\,:\,\varrho_i = \mathrm{Ob}} S(\boldsymbol{x}, \boldsymbol{x}_i) \quad \text{for} \ \boldsymbol{x} \in B(t)\,, \tag{5}$$

where function S specifies the rate of secretion at position $\boldsymbol{x}$ due to an osteoblast at positions $\boldsymbol{x}_i$. As osteoblasts can only secrete bone locally, the function $S(\boldsymbol{x}, \boldsymbol{y})$ decays for $||\boldsymbol{x} - \boldsymbol{y}|| \gg \iota$ for some characteristic length scale ι. For simplicity, we choose the function

$$S(\boldsymbol{x}, \boldsymbol{y}) = \eta \, \exp\left(-\frac{1}{2}\iota^{-2}||\boldsymbol{x} - \boldsymbol{y}||^2\right) . \tag{6}$$

(ii) An osteoblast with degree k (or k dendrite structures attached to it), differentiates into an osteocyte at rate D_k.The precise choice of the form of D_k is discussed in Section **??**.

(iii) Connections are created from osteocytes in $\Omega(t)$ towards the osteoblast layer $B(t)$. We specify that osteocytes at position $\boldsymbol{x} \in \Omega(t)$ can create connections to osteoblasts at position $\boldsymbol{y} \in B(t)$ at rate $\mathcal{C}(\boldsymbol{x}, \boldsymbol{y})$. We make the assumption that the rate is proportional to a Gaussian with the argument of distance between $\boldsymbol{x}$ and $\boldsymbol{y}$. An connection is only able to form provided there exists a straight line path within the bone domain $\Omega(t)$ between both nodes. Therefore

$$\mathcal{C}(\boldsymbol{x}, \boldsymbol{y}) = \begin{cases} \alpha \, \exp\left(-\frac{1}{2}\beta^{-2}||\boldsymbol{x} - \boldsymbol{y}||^2\right) & (\boldsymbol{x}, \boldsymbol{y}) \in P\,, \\ 0 & (\boldsymbol{x}, \boldsymbol{y}) \notin P\,, \end{cases} \tag{7}$$

for constants $\alpha, \beta > 0$, and

$$P := \{(\boldsymbol{x}, \boldsymbol{y}) \in \Omega \times \Omega \,|\, s\boldsymbol{x} + (1 - s)\boldsymbol{y} \in \Omega \ \text{for} \ s \in [0, 1]\} . \tag{8}$$

(iv) Osteoblasts disconnected from the network (osteoblasts of degree 0) are allowed to move on the bone surface manifold $B(t)$. We model this movement using Brownian motion on the manifold $B(t)$ with corresponding diffusion constant $\kappa_{\mathrm{diff}} > 0$.

(v) Osteoblasts disconnected from the network (osteoblasts of degree 0) are allowed to proliferate on the bone surface manifold $B(t)$. As we are not modelling finite volume effects, the new daughter osteoblast will be positioned at the same position as the parent cell. We model this proliferation as a time dependent growth rate $\mu(t)$.

Table 2. Model functions.

Function name	Symbol and form	Units	Comment
Rate of dendrite growth	$\mathcal{C}(\boldsymbol{x}, \boldsymbol{y}) = \alpha \, \exp\left(-\frac{1}{2}\beta^{-2}\|\|\boldsymbol{x} - \boldsymbol{y}\|\|^2\right)$	day^{-1}	See Equation (7). Assumption that ability to dendrites from osteocyte to osteoblast is normally distributed.
Rate of bone secretion	$S(\boldsymbol{x}, \boldsymbol{y}) = \eta \, \exp\left(-\frac{1}{2}\iota^{-2}\|\|\boldsymbol{x} - \boldsymbol{y}\|\|^2\right)$	$\mathrm{mm}\,\mathrm{day}^{-1}$	See Equation (5).
Initial condition for Ob. surface den.	$v_k(t = 0) = \tilde{p}\mathbb{1}[\boldsymbol{x} \in \{\omega_0\} \times (0, L_y)]\delta_{k,0}$	mm^{-2}	–
Initial condition for Ocy. number den.	$w_k(t = 0, \boldsymbol{x}) \equiv 0$	mm^{-3}	–

Table 3. Choice in rate of osteoblast differentiation.

<table>
<tr><th>Symbol and form</th><th>Interpretation</th></tr>
<tr><td>$D_k^{(\text{null})} = \hat{D}$</td><td>No network effects.</td></tr>
<tr><td colspan="2">This corresponds to the null hypothesis: the network does not impact the rate of osteoblast differentiation.</td></tr>
<tr><td>$D_k^{(\text{swt})} = \begin{cases} \lambda_{\text{swt}} & \text{if} \quad k = 0\,, \\ \lambda_{\text{swt}} + \gamma_{\text{swt}} & \text{if} \quad k \geq 1\,. \end{cases}$</td><td>Switch-like influence.</td></tr>
<tr><td colspan="2">The network does impact osteoblast differentiation rates but there are no cumulative effects, i.e., communicating with more osteocyte does not mean osteoblasts differentiate any faster/slower.</td></tr>
<tr><td>$D_k^{(1)} = \lambda_1 + \gamma_1 k$</td><td>Cumulative activation.</td></tr>
<tr><td colspan="2">The osteoblast will differentiate at a rate that increases proportional to how many osteocytes it is in communication with.</td></tr>
<tr><td>$D_k^{(2)} = \begin{cases} \lambda_2 & \text{if} \quad k = 0\,, \\ \lambda_2 + \gamma_2/k & \text{if} \quad k \geq 1\,. \end{cases}$</td><td>Cumulative inhibition.</td></tr>
<tr><td colspan="2">The osteoblast will differentiate at a rate that increases inversely proportional to how many osteocytes it is in communication with.</td></tr>
</table>

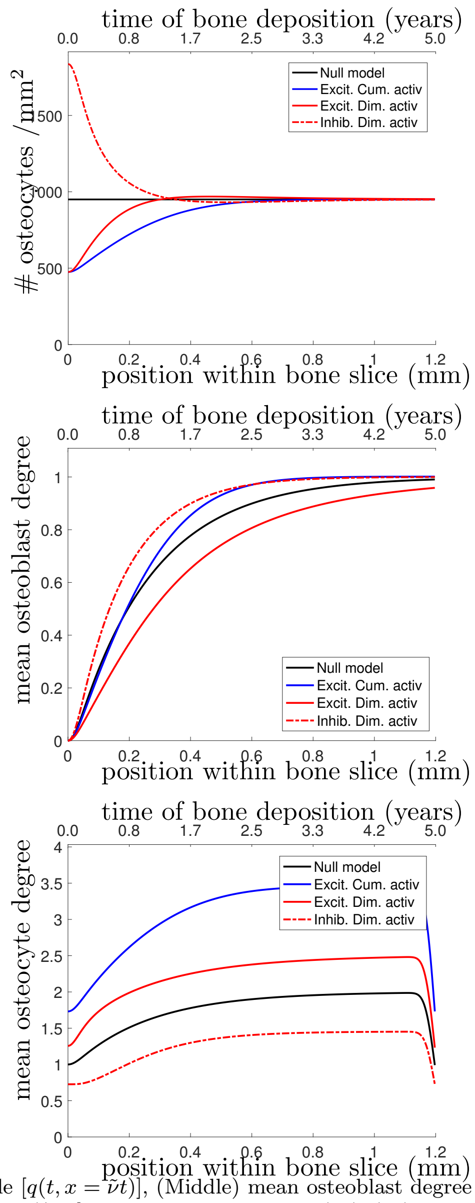

Fig 4. (Top) Osteocyte density profile $[q(t, x = \tilde{\nu}t)]$, (Middle) mean osteoblast degree over time $[\langle k(t = \tilde{\nu}t)\rangle_{\mathrm{Ob}}]$, and (Bottom) mean osteocyte degree over time $[\langle k(t = \tilde{\nu}t)\rangle_{\mathrm{Ot}}]$ when solving equations (11)–(12). The black line shows the null model, the blue line shows the excitatory cumulative activation model, and the red lines shows the diminishing activation model, excitatory effects using the solid line and inhibitory effects using the dot-dashed line.
